# Ruminal bacterial communities differ in early-lactation dairy cows with differing risk of ruminal acidosis

**DOI:** 10.3389/frmbi.2023.1212255

**Published:** 2023-09-29

**Authors:** Helen Marie Golder, Josh Rehberger, Alexandra Helena Smith, Elliot Block, Ian John Lean

**Affiliations:** ^1^ Scibus, Camden, NSW, Australia; ^2^ Dairy Science Group, School of Life and Environmental Sciences, Faculty of Science, The University of Sydney, Camden, NSW, Australia; ^3^ Arm & Hammer Animal and Food Production, Princeton, NJ, United States

**Keywords:** crude protein, diet nutrients, microbiota, ruminal acidosis, sugar

## Abstract

**Introduction:**

Early-lactation Holstein cows (*n*= 261) from 32 herds in three regions (Australia, California, and Canada) were previously categorized using a discriminant analysis model as being at a high (26.1% of cows), medium (26.8% of cows), or low risk (47.1% of cows) of ruminal acidosis. We aimed to investigate if (1) risk of acidosis would be associated with ruminal bacterial taxa and dietary nutrient components, (2) there would be individual or combinations of bacterial taxa associated with acidosis-risk groups, and (3) the abundance of bacterial taxa would be associated with the intake of dietary nutrient components.

**Methods:**

Diets ranged from pasture supplemented with concentrates to total mixed rations. Bacteria 16S ribosomal DNA sequences from rumen samples collected < 3 hours after feeding via stomach tube were analyzed to determine bacterial presence. The relative abundance of each bacterial phylum and family was center log transformed and the transformed family data were subjected to two redundancy analysis biplots, one for acidosis risk group and one for region, to identify the 20 best-fit bacterial families from each respective redundancy analysis. A total of 29 unique families were identified when the lists of 20 families were combined from each redundancy analysis, and these 29 families were termed "influential" families." The association of acidosis-risk groups with the abundance of individual influential families was assessed by mixed models. Backward stepwise elimination mixed models were used to determine the bacterial taxa associated with each acidosis-risk group and the dietary nutrients associated with the abundance of the bacterial taxa.

**Results and discussion:**

High-risk acidosis cows were associated with increased abundances of Anaerocella_f and Veillonellaceae and decreased abundances of several bacterial families with different characteristics. Five phyla: Firmicutes [odds ratio (OR) = 7.47 ± 7.43], Spirochaetes (OR = 1.28 ± 0.14), Lentisphaerae (OR = 0.70 ± 0.07), Planctomycetes (OR = 0.70 ± 0.09), and Tenericutes (OR = 0.44 ± 0.15), and nine families were associated with a higher risk of acidosis. Of the nine phyla identified to be of interest based on abundance and strength of association with acidosis-risk groups, all had one or more dietary nutrient that predicted their abundance. Sugar was the most frequently associated nutrient with the nine phyla, and was present in 78% (seven out of nine phyla) of the models; crude protein was present in 56% of models and crude fat was present in 44% of the models. Sugar and crude protein were most associated with the influential families and all but three families had one or more nutrient predictive of their abundance. Ruminal bacterial taxa are associated with ruminal acidosis; dietary sugar and crude protein are vital predictors of these and, thus, of ruminal acidosis risk.

## 1 Introduction

The development of feed management practices that promote optimal production efficiency requires a deep understanding of the complex and dynamic rumen microbiome (Fernando et al., 2010; de Menezes et al., 2011), which substantially influences metabolism in ruminants (Shabat et al., 2016). Ruminal acidosis is an important example of an interaction between the rumen microbiome and diet that can impair health and production (Tajima et al., 2000; Khafipour et al., 2009). The type of dietary substrate, method of processing, and time of availability relative to other substrates are among the risk factors for acidosis that can be managed (Lean et al., 2014). Dietary physically effective neutral detergent fiber that increases milk fat percentage, rumen pH, and fermentation (Allen, 1997; Mertens, 1997; Zebeli et al., 2012; Nasrollahi et al., 2017) also influences the risk of acidosis. Bramley et al. (2008) found that herds containing a higher prevalence of cattle with ruminal acidosis were fed diets that were high in non-fiber carbohydrates and low in neutral detergent fiber (NDF).

Henderson et al. (2015) postulated the presence of core microbiota that reflect relatively similar abundances of rumen bacteria in a range of ruminants, diets, and feeding strategies; however, bacteria have different substrate requirements and relationships (Hungate, 1966; Kamra, 2005). Much of the work to date has examined the abundances and function of ruminal bacterial taxa independent of other bacterial populations, and features a limited exploration of associations among these and does not provide detailed dietary descriptions, metabolomics, and production outcomes. There is strong interdependency among bacteria within rumen biofilms (Leng, 2018) and the role of the exometabolome (Douglas, 2020) gives support to the concept of the “Black Queen Hypothesis” of bacteria inter-dependencies (Morris et al., 2012) being present in the rumen. Consequently, there is a need for a more comprehensive approach to evaluating the rumen. Advances in technology and analytical processes have produced an increase in the number of metagenomic approaches available and provide increased potential to achieve this. Henderson et al. (2015)reported that microbial interactions were not exclusive associations, with relatively few co-occurrence patterns. There is an opportunity to identify the key microbiota associated with the aetiology and control of acidosis and to determine if they can be used to effectively define, predict, and prevent acidosis. Although this is not currently practical in the field, the knowledge of which taxa are associated with ruminal acidosis and their associations with dietary nutrients can aid nutritionists and managers in diet formulation and in the implementation of preventive management strategies, and guide the direction of future research.

The focus of definitions for ruminal acidosis has shifted from rumen pH to the rumen microbiome and its metabolic activity (Bramley et al., 2008; Saleem et al., 2012; McCann et al., 2016). Plaizier et al. (2018) suggest that an accurate diagnosis of ruminal acidosis requires a combination of clinical examinations of cows and analyses of herd management and feed quality, accompanied by rumen pH, blood, milk, urine, and fecal parameters.

Bramley et al. (2008) created a model based on a K-Means cluster and discriminant analysis that classified 800 cows from 100 randomly selected commercial dairy herds across South Australia based on their rumen pH, individual volatile fatty acids (VFA), ammonia, and D-lactate concentrations into one of three categories (1) acidotic, (2) suboptimal rumen function, or (3) normal (Bramley et al., 2008; Golder et al., 2012a). Golder et al. (2023b)used the model by Bramley et al. (2008) to classify 293 early lactation Holstein cattle from four geographical regions into three acidosis risk groups (high, medium, and low). The overall bacterial community composition differed among acidosis groups and regions and bacterial community composition was found to be associated with rumen metabolites and milk production (Golder et al., 2023b). The abundance of individual ruminal bacterial phyla also differed among acidosis risk groups, but exploration ruminal bacteria at lower taxonomic levels is required.

This paper builds on the observations of Golder et al. (2023b)and Golder et al. (2023c) to improve our understandings of acidosis risk and, ultimately, to control ruminal acidosis through the evaluation of its associations with host genomics, diet, rumen metabolites, ruminal bacterial taxa, and production characteristics in early-lactation Holsteins. We hypothesized that (1) acidosis risk would be associated with ruminal bacterial taxa and dietary nutrient components, (2) there would be individual or combinations of bacterial taxa that would be associated with acidosis risk groups, and (3) the relative abundance (RA) of bacterial taxa would be associated with the intake of diet nutrient components.

## 2 Methods

This study was approved by the S*cibus* Animal Ethics Committee (Scibus 0618-1219); the University of California Davis Institutional Animal Care and Use Committee (protocol number 20729); the University of Wisconsin, College of Agriculture and Life Sciences Animal Care and Use Committee (approval A006225); and the Animal Care Committee at the University of Guelph (Animal Utilization Protocol 4124). 

### 2.1 Experimental design

This was a multicenter observational study. The study population comprised 293 cows from 36 herds [10 in California, USA (CA); 12 in Canada (CAN); 10 in Australia (AU); and 4 in Wisconsin, USA (WI)], with a target of eight cows per herd (four primiparous and four of parity >1 and ≤4) between 10 and 100 DIM. The AU herds were in four geographical regions (the Western districts of Victoria, Finley in NSW, the South Coast of NSW, and the Macarthur region of NSW). The herds ranged in size from 57 to 6,294 cows and are summarized by Golder et al. (2023b). The diets of the North American herds were all total mixed rations, whereas those in Australia were primarily pasture-based with supplementary concentrates and/or silage.

### 2.2 Power calculations

Power calculations were carried out using the *rdpower* function in Stata (version 14.2; StataCorp LLC, College Station, TX) and were based on a difference of 5.5 m*M* in the VFA propionate, measured with a SD of 10 m*M* being sufficient to categorize non-acidotic cows (Bramley et al., 2008). Consequently, a difference of 29m*M*–34.5 m*M* in propionate would require 320 cows, given an intraclass correlation of 0.2. The study operated on two levels. The effect size (ES) was 0.55, the number of head per herd was eight (*n*), and the number of herds was 40 (m) to provide a power of 0.6.

### 2.3 Farm and cow selection

Farms were selected based on their willingness to participate and if they had accurate details on cow identification, parentage, and calving history; predominately Holstein-Friesian lactating cattle; conducted herd recording; and were able to provide diet details and a sample of the diet for analysis. There were no restrictions based on herd size, production level, production system, or milking frequency per day.

Cows that met the following criteria were randomly selected: they were Holstein-Friesian cows, were fourth-parity cows at maximum (four cows of parity 1, and four cows of parity >1 and ≤4), were between 10 and 100 DIM, had no apparent current clinical illness, and were from different sires. This information was obtained using each herd’s herd management software.

### 2.4 Sample collections and analysis

Sample collections included rumen fluid for ruminal metabolomic analysis, bacterial taxonomy analysis, and determination of acidosis risk group; milk samples for production data; and samples of the diet for analysis of nutrient composition. Detailed descriptions of collection and analyses are provided by Golder et al. (2023b).

#### 2.4.1 Rumen metabolomics

Up to 500 mL of rumen fluid was collected via a stomach tube within 3 hours of feeding. Each sample was tested for saliva in accordance with the method described by Golder et al. (2023b) and Bramley et al. (2008) and a pH measurement was taken immediately (LAQUAtwin pH-22, Horiba, Kyoto, Japan). A 10-mL aliquot of raw rumen fluid was placed on ice prior to being centrifuged at 1,110 g for 15 minutes, and the supernatant was aliquoted for analysis. Rumen VFA concentrations were analyzed using gas chromatography (Supelco, 1975). Ammonia concentrations were analyzed using a direct enzymatic method utilizing the Beckman Coulter Reagent (Category no OSR61154; Lane Cove West, NSW, Australia). Lactate concentrations were analyzed after protein removal using perchloric acid precipitation. An enzymatic sample blanked end-point assay was used incorporating glutamate-pyruvate transaminase and either L-lactate dehydrogenase (Roche Holding AG; Basel, Switzerland) or D-lactate dehydrogenase (Megazyme catalog no. E-DLDHLM; Wicklow, Ireland).

#### 2.4.2 Acidosis risk group

The acidosis risk group for each cow at the time of rumen sampling was determined by incorporating their individual VFA, ammonia, total lactate, and pH values into the existing data set by Bramley et al. (2008), which was used to develop the discriminant analysis and K-Means clusters. A discriminant analysis was used (StataCorp. version 16.1, TX) to classify each of the cows in this study into one of the three acidosis categories defined by K-means clustering by Bramley et al. (2008). As the characteristics of our categories were likely to differ to those of Bramley et al. (2008), to distinguish them we termed our categories as ‘high’, ‘medium’, and ‘low’ risk for ruminal acidosis. The distance to the centroid for each of the three known acidosis risk groups (high, medium, and low) was produced, where values approaching 1 were in the center of the group.

#### 2.4.3 Ruminal bacterial taxonomy

Samples (2 × 15 mL) of raw rumen fluid were immediately stored on ice after collection, prior to storage at −20°C, and later shipped on dry ice to Arm & Hammer Animal and Food Production (Waukesha, WI, USA) for genomic DNA extraction using the DNeasy PowerSoil HTP Kit (QIAGEN, Germantown, MD, USA). Genomic DNA was shipped to the Roy J. Carver Biotechnology Center (The University of Illinois at Urbana-Champaign, Champaign, IL, USA) for library preparation and sequencing. PCR was conducted for 35 cycles with 16S V4 rRNA primers for total bacteria (Walters et al., 2016) using the 48 Access Array IFC (Fluidigm, San Francisco, CA, USA). The libraries were sequenced from both ends of the molecules to a total read length of 250 nt from each end using NovaSeq 6000 (Illumina Inc., San Diego, CA, USA).

Sequence analysis was conducted using QIIME2 version 2021.2 (Bolyen et al., 2019). Quality filtering was carried out to remove any sequences with a Phred quality score below 22 (Bokulich et al., 2013). Sequences were denoised and trimmed to 240 base pairs using deblur (Amir et al., 2017). Taxonomy was assigned by closed-reference clustering using VSEARCH (Rognes et al., 2016) at an operational taxonomic unit level of 97% to the EZBioCloud reference database (Kim et al., 2012), downloaded 15 May, 2017. All samples with fewer than 7,000 reads were removed, and all Archaea and Eukarya were removed from the analysis. The remaining samples had an average of 21,065 reads with a standard deviation of 10,776 reads. The median number of reads in remaining samples was 18,948, with the 10th percentile being 9,223 reads, and the 90th percentile being 36,366 reads. Rarefaction analysis (Hughes et al., 2001) indicated that the depth of coverage of diversity of rumen bacteria within the rumen fluid samples was sufficient to evaluate bacteria community composition (Golder et al., 2023a; Golder et al., 2023b).

Beta diversity was examined using Canoco5 (Microcomputer Power, Ithaca, NY), a multivariate statistical analysis program used in ecology (Lepš and Šmilauer, 2003). Count tables were log-transformed and centered by bacterial family and a redundancy analysis (a linear, constrained ordination method) was carried out. Constrained ordination is a way to relate multiple variables (e.g., bacterial families) to explanatory variables (e.g., acidosis risk group, region, and dietary nutrients). Redundancy analysis is used to visually represent the differences among samples, but similar to PCA, can also show the values of the bacterial families fitted. Data are shown as a biplot generated by Canoco5 with the 20 bacterial families that had the best-fit for acidosis risk group, region, and dietary nutrients. The amount of variation in the model, for each axis, and for each explanatory variable included was calculated. The model was run with data from all 36 farms from the four regions initially, but the final model included data from 32 farms from three regions only, to provide consistency with the data set used in the mixed-model analysis. The significance of each explanatory variable was determined by a Bonferroni corrected *p*-value.

#### 2.4.4 Nutrient composition of diet

A sample of total mixed ration (approximately 500 g) was collected from each of the herds in California and Canada, and from one in Wisconsin, and these were analyzed using wet chemistry at DairyOne Cooperative Inc., Forage Testing Laboratory (Ithaca, NY, USA) in accordance with the wet chemistry AOAC International (1999) methods detailed in Golder et al. (2019). For the herds in Australia, ~ 500 g of feed components were analyzed using the methods described in Golder et al. (2023b). The diet chemical composition for these herds was then estimated using CPM Dairy Ration Analyzer (version 3.10; Cornell-Penn-Miner, Cornell University). For three herds from Wisconsin, their nutritionist provided details of the ration components and respective feed tests of these components for an estimation of diet chemical composition in CPM Dairy Ration Analyzer. The key nutrient composition measures for each herd are reported by Golder et al. (2023b). The mean ± SD for each region and the mean ± SD of all regions combined are presented in **Table 1**.

**Table 1:** Mean ± SD for key diet nutrients for each region and total of all regions (% of DM).

Region	Number of farms	CP	ADF	NDF	NFC	Starch	Sugar	Crude fat
Australia	10	19.4 ± 3.95	21.8 ± 3.96	37.1 ± 6.40	33.7 ± 5.26	19.8 ± 4.70	6.32 ± 2.26	4.24 ± 0.52
California	10	16.2 ± 0.79	21.7 ± 1.35	29.5 ± 1.69	42.6 ± 2.48	22.4 ± 2.11	7.17 ± 1.80	4.90 ± 0.68
Canada	12	16.3 ± 1.05	22.9 ± 2.85	34.0 ± 5.44	38.3 ± 7.35	23.4 ± 7.63	3.54 ± 1.08	5.47 ± 2.71
Wisconsin	4	17.6 ± 0.54	19.6 ± 1.74	36.8 ± 14.1	42.3 ± 0.72	27.0 ± 1.88	4.01 ± 0.93	4.71 ± 0.58
All regions		17.3 ± 2.56	21.9 ± 2.87	33.9 ± 6.85	38.6 ± 6.22	22.5 ± 5.48	5.37 ± 2.27	4.88 ± 1.66
Range		14.5 – 26.5	16.1 – 29.7	27.2 – 58.0	17.0 – 46.7	0.10 – 29.8	2.10 – 11.2	3.50 – 13.5

### 2.5 Statistical analysis

#### 2.5.1 Determining influential families

**Figure 1:**
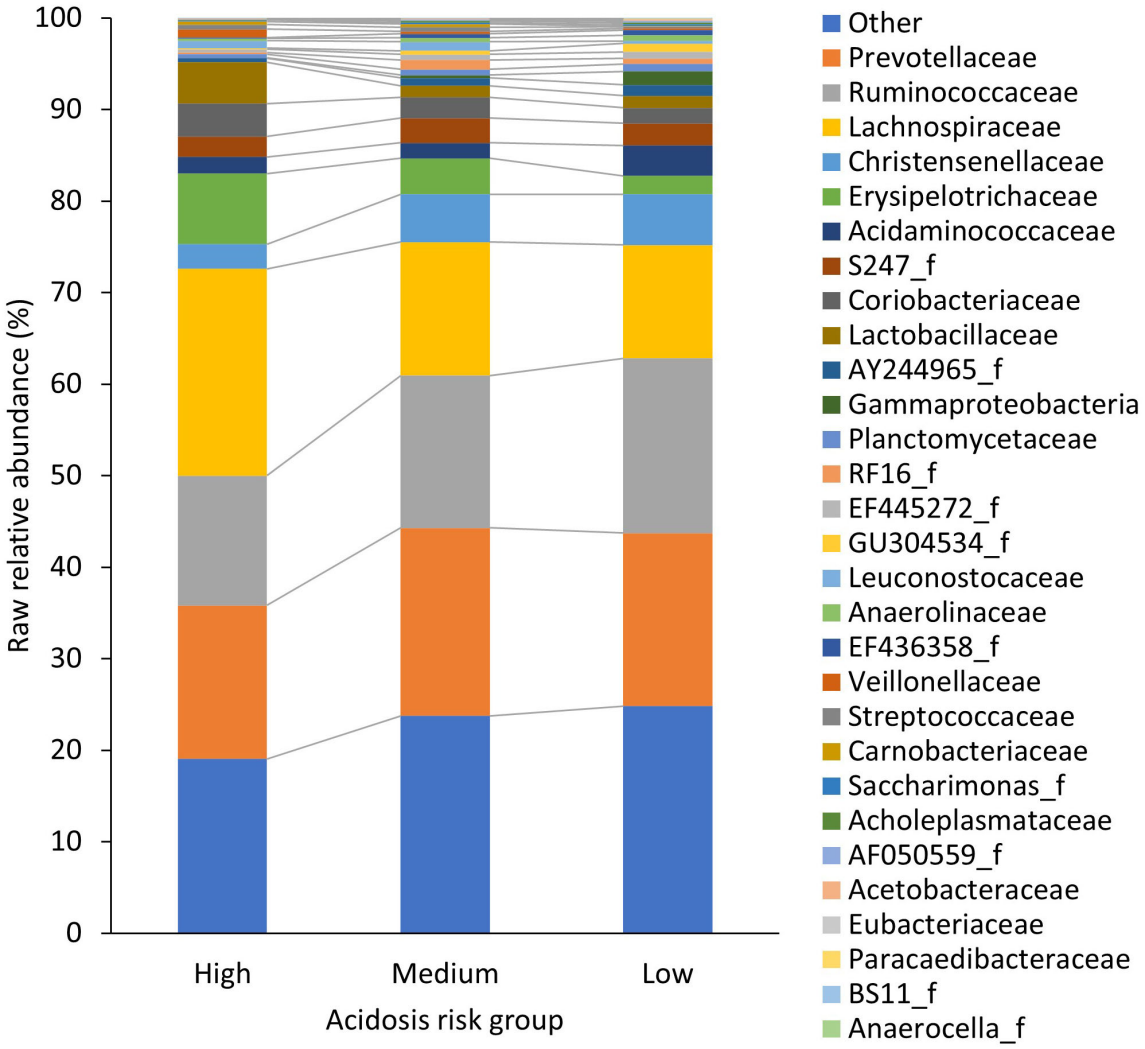
The raw relative abundance (%) of ruminal bacterial families in each acidosis risk group. Only the 29 families that made up the “influential” families, which are an amalgamation of the 20 families that had the best-fit from a redundancy analysis of acidosis risk group and a second redundancy analysis of region, are displayed. The raw relative abundance of all other phyla (*n* = 170) was combined and displayed as “other”, along with sequences that could not be classified.

To determine which of the identified 199 families may have had the greatest association with acidosis risk group, region, and dietary nutrients, and to allow a more in-depth exploration of these, redundancy analysis in Canoco5 (Microcomputer Power) was used. The 20 families with the best fit from two redundancy analysis biplots (similar to constrained PCA plots), one for acidosis risk groups (**Supplementary Figure 1**) and one for region (**Supplementary Figure 2**), were amalgamated into a list of 29 different families and were termed “influential” families (11 families were in both biplots). The “best fit” metric is equivalent to the percentage of variation explained. Specifically, we used the “CFit2” metric from Canoco5, which represents the cumulative fraction of variation of individual families across the *x* and *y* ordination axes. The *x*and *y*coordinates and Cfit2 values for the 20 families with best fit for (1) acidosis risk group and (2) region are shown in **Supplementary Table 1**. The mean RAs of the 29 influential families by acidosis group are displayed as a stacked column graph (**Figure 1**). The raw RAs of all other families (*n*= 170) were combined and displayed as “other”, along with sequences that could not be classified. Data are from 32 farms across three regions.

#### 2.5.2 Prevalence of bacterial taxa per group

The RA of each sequenced bacterial phylum and family in one rumen fluid sample per cow were center log transformed (CLT) in Stata version 16.1 (Statacorp) prior to further analysis. To assess the association of acidosis-risk groups with the abundance of individual rumen bacterial families that were identified as “influential” in redundancy analyses, a mixed model with the fixed effects of acidosis group, parity, and region, and their interactions with the random effects of herd nested within region, was used (Stata). This is the same model that was used by Golder et al. (2023b) to assess the association of acidosis group with the probability of distance to the centroid of each of the acidosis groups, rumen metabolites, abundance of rumen bacterial phyla, and milk production. The data from the four herds in Wisconsin were removed from this dataset as the selection criteria for the percentage of parity-1 cows sampled were not met. The model was as follows:

Y_ijklm_= µ + α_i_+ γ_j_+ θ_kl_+ *ωα*γθ_ijk_+ f_k_+ r_i:mj_+ c_l:ijkm_+ ϵ_ijklm_

Y_ijklm_= µ is the overall mean; α_i_ is the fixed effect of acidosis group (i = group 1, 2, or 3), γ_j_ is the is the fixed effect of parity (j = primiparous or multiparous); θ_kl_ is the fixed effect of region (k = AU, CA, and CAN) for cow number l (l = 1 to a maximum of 261); *ωα*γθ_ijk_ are the fixed effects of interaction terms, comprising two- and three-way interactions of acidosis group, parity, and region; f_k_ is the random effect of region; r_i:mj_ is the random effect for the i^th^ acidosis group within the m^th^ herd and the k^th^ region; c_l:ijkm_ is the random interaction effect associated with the l^th^ cow with the j^th^ parity within the i^th^ acidosis group within the m^th^ herd and the k^th^ region; and ϵ_ijklm_ is the random error term. The covariance was unstructured.

#### 2.5.3 Associations of group with bacterial taxa

To determine the bacterial taxa associated with each acidosis group, backward stepwise elimination mixed models with a significance of *p*< 0.05 using the *melogit* function in Stata were applied. Models included the fixed effects of herd nested within region and were run separately at the phylum and family level and for each acidosis risk group, providing six elimination models. All 36 herds from the four regions were included. To screen the large number of bacterial taxa prior to inclusion in the models, univariable logistic regression was conducted using the *melogit* function for each of the CLT abundances of phyla and families for each of the acidosis groups. No fixed effects were included in these univariable models. All phyla and families that were significant at *p*< 0.100 after univariable logistic regression analysis were entered into the backward stepwise models. Odds ratios of the CLT abundances were determined after completion of the stepwise elimination. To determine each model’s performance in distinguishing between positive and negative cases, an area under the curve was calculated using the *predict* and *roctab* functions in Stata.

#### 2.5.4 Prediction of abundance of bacterial taxa by dietary nutrient component

Backward stepwise elimination mixed models (Stata *mixed*) were used to determine which dietary nutrients were associated (*p*< 0.100) with the CLT of abundance of the bacterial taxa. Region was included in the model, and data from all 36 herds from the four regions were used. The dietary nutrients expressed as percentages of dry matter included in the model were: NDF, crude protein (CP), starch, sugar, and crude fat. If confounding occurred, as indicated by a 20% change in co-efficient for another nutrient, the non-significant nutrient was also retained in the model. Separate models were run for each of the 26 bacterial phyla that had at least one sample with a relative abundance of > 0.3%, and also for each of the 29 “influential” families identified as the best fit in the redundancy analysis. To determine each model’s performance, the root mean square error was calculated using the *regress* function in Stata, with region removed from the model.

## 3 Results

A total of 199 families were identified. Overall, Prevotellaceae was the most dominant family, followed by Ruminoccoceae and Lachnospiraceae; the associated mean raw RAs were 19.1% ± 13.6, 16.9% ± 7.5, and 15.8% ± 8.9 per cow, respectively. The bacterial family in the high-risk acidotic group with the highest RA was Lachnospiraceae (**Figure 1**). In the medium-risk group, excluding “other” bacteria, the most dominant family was Prevotellaceae and in the low-risk group, also excluding “other” bacteria, it was Ruminococcaceae (**Figure 1**). All abundances reported hereafter are center-logged ratios that were the product of the CLT of the raw RA of the 29 “influential” families. Descriptions of these taxa are presented in **Table 2**.

**Table 2:** The lineage, Gram stain status, and description of characteristics of the 29 families that were considered as the 20 “influential” families with the best-fit for either acidosis risk group or region based on redundancy analyses.

Lineage	Gram stain (+/−)	Description of characteristics
Actinobacteria, Coriobacteria, Coriobacteriales, Coriobacteriaceae	+	Strictly anaerobic bacteria can be considered as pathobionts, many species are asaccharolytic (Clavel et al., 2014)
Bacteroidetes, Bacteroidia, Bacteroidales, Anaerocella_f	−	Strict anaerobe that does not utilize carbohydrates or organic acids, weak utilization of peptone and some amino acids, produces VFA (Abe et al., 2012)
Bacteroidetes, Bacteroidia, Bacteroidales, AY244965_f	−	The order Bacteroidalesare mostly anaerobic and saccharolytic but some can utilize proteins and other substrates (Krieg, 2010)
Bacteroidetes, Bacteroidia, Bacteroidales, BS11_f	−	BS11_f have multiple pathways for degrading hemicellulose sugars to short-chain fatty acids, i.e., butyrate and acetate (Solden et al., 2017)
Bacteroidetes, Bacteroidia, Bacteroidales, GU304534_f	−	The order Bacteroidalesare mostly anaerobic and saccharolytic but some can utilize proteins and other substrates (Krieg, 2010)
Bacteroidetes, Bacteroidia, Bacteroidales, Prevotellaceae	−	Anaerobic and saccharolytic (Krieg, 2010)
Bacteroidetes, Bacteroidia, Bacteroidales, RF16_f	−	The order Bacteroidalesare mostly anaerobic and saccharolytic but some can utilize proteins and other substrates (Krieg, 2010)
Bacteroidetes, Bacteroidia, Bacteroidales, S24-7_f	−	Carbohydrate fermenters or nanaerobic, producing acetate, propionate, and succinate, maybe targeted by the innate immune system (Ormerod et al., 2016)
Chloroflexi, Anaerolineae, Anaerolinaeles, Anaerolinaceae	−	Variable use of monosaccharides, pyruvate and starch depending on species, optimal growth at pH 7 and no use of lactate (Yamada et al., 2006)
Firmicutes, Bacilli, Lactobacillales, Carnobacteriaceae	+	Most species are facultatively anaerobic lactic acid producing bacteria that ferment pentose and hexoses. They also produce CO_2_, ethanol, and acetate (Bergey et al., 2011)
Firmicutes, Bacilli, Lactobacillales, Lactobacillaceae	+	Fermentative metabolism, obligately saccharo-clastic bacteria that produce half of their end product as lactate. Acetate, ethanol, CO_2_, formate, or succinate may be produced (Bergey et al., 2011)
Firmicutes, Bacilli, Lactobacillales, Leuconostocaceae	−	Facultatively anaerobic, heterofermentatively ferment glucose to lactate, CO2, acetate, and ethanol (Bergey et al., 2011)
Firmicutes, Bacilli, Lactobacillales, Streptococcaceae	+	Facultatively anaerobic, ferment carbohydrates to mainly lactate, some produce bacteriocins (Bergey et al., 2011)
Firmicutes, Clostridia, Clostridiales, Christensenellaceae	+	Anaerobic and utilize various sugars to produce VFA (Morotomi et al., 2012)
Firmicutes, Clostridia, Clostridiales, Eubacteriaceae	+	Chemo-organotrophs, many ferment sugars and utilize proteinaceous nitrogen sources. Mesophilic to moderately thermophilic and neutrophilic to alkaliphilic with some halotolerant species (Bergey et al., 2011)
Firmicutes, Clostridia, Clostridiales, Lachnospiraceae	+	Anaerobic and morphologically diverse, ferment a diverse range of polysaccharides to butyrate and other short chain fatty acids (Bergey et al., 2011)
Firmicutes, Clostridia, Clostridiales, Ruminococcaceae	+	Obligate anaerobes with diverse morphology, ferment carbohydrates to VFA, lactate, and ethanol. Some utilize H_2_ (Bergey et al., 2011)
Firmicutes, Erysipelotrichia, Erysipelotrichales, Erysipelotrichaceae	+	Aerobic to facultatively anaerobic, chemo-organotrophic, with respiratory metabolism and are weakly fermentative. Produce acid but no gas from glucose and other carbohydrates (Bergey et al., 2011)
Firmicutes, Negativicutes, Acidaminococcales, Acidaminococcaceae	+	Asaccharolytic and growth not observed with succinate but can produce propionate (Marchandin et al., 2010). Is a D-lactate producer (Al Jassim et al., 2005)
Firmicutes, Negativicutes, Veillonellales, Veillonellaceae	−	Formerly “Acidaminococcaceae”. Obligate anaerobes with diverse characteristics, including lactate utilization (Bergey et al., 2011)
Lentisphaerae, CP010904, EF436358, AF050559_f	−	The class CP010904 metabolizes sulfated glycopolymers and monosaccharides but only low amounts of glucose which has the end products of ethanol, acetate and lactate (Spring et al., 2016)
Lentisphaerae, CP010904, EF436358, EF436358_f	−	
Planctomycetes, Planctomycetia, Planctomycetales, Planctomycetaceae	−	Can utilize carbohydrates and hydrogen, can synthesize amino acids, unsaturated fatty acids and complex lipids such as steroids. Can generate energy through sulfur and nitrogen (Guo et al., 2014)
Proteobacteria, Alphaproteobacteria, Rhodospirillales, Acetobacteraceae	−	Acetic acid, acidophilic, phototrophic, obligately aerobic bacteria (Kersters et al., 2006). Known as vinegar-producing bacteria
Proteobacteria, Alphaproteobacteria, Rickettsiales, Paracaedibacteraceae	−	Aerobic and mesophilic (Hess et al., 2016)
Proteobacteria, Gammaproteobacteria, Gammaproteobacteria_c	−	Large number of genera with diverse morphology, aerobicity, and trophism. Includes many deleterious species (Williams et al., 2010)
Saccharibacteria, Saccharimonadia, Saccharimonadales, Saccharimonadaceae	+	Saccharibacteria uses only the pentose phosphate and heterolactic fermentation pathways, converting glucose to lactate and acetate and has tolerance of oxygen (Albertsen et al., 2013)
Tenericutes, Mollicutes, Acholeplasmatales, Acholeplasmataceae	−	Saprophytic bacteria, the family is characterized by a lack of sterol requirement for growth (Siewert et al., 2014)
Tenericutes, Mollicutes, AM275436, EF445272_f	−	The order Mollicutes are pleomorphic, small prokaryotes with no cell wall, most species are facultative anaerobes, many are pathogens (Boone et al., 2011)

### 3.1 Bacterial prevalence

In the high-risk group, the abundances of Anaerocella_f and Acholeplasmataceae were increased, whereas those of AY244965_f, Anaerolineaceae, Christensenellaceae, Ruminococcaceae, AF050559_f, EF445272_f, and Planctomycetaceae were decreased compared with the medium- and low-risk groups, which had similar abundances (**Table 3**). The abundance of Gammaproteobacteria_c was lowest, whereas that of Veillonellaceae was greatest in high-risk cows, followed by cows of low-risk and then medium-risk. Low-risk cows had an increased abundance of GU304534_f, EF436358_f, and Paracaedibacteraceae and a decreased abundance of Lachnospiraceae, compared with the other groups, which had similar abundances. The abundances of BS11_f and Erysipelotrichaceae were also greater in low-risk cows than in high-risk cows, but similar in high- and medium-risk cows, and also in medium- and low-risk cows. Medium-risk cows had greater abundances of RF16_f and Saccharimonadaceae.

**Table 3:** The mean ± SE of the center-logged ratio relative abundances of ruminal bacterial families for each acidosis risk group and significances^1^.

Phyla	Family	Gram stain (+/-)	Acidosis risk group			*p*-value						
			High	Medium	Low	Group (G)	Region (R)	Parity (P)	G × R	G × P	R × P	G × R × P
Actinobacteria	Coriobacteriaceae	+	0.21 ± 0.238	0.18 ± 0.187	−0.20 ± 0.155	0.108	<0.001	0.305	0.931	0.846	0.916	0.909
Bacteroidetes	Anaerocella_f	−	0.93^a^± 0.209	−0.14^b^± 0.157	−0.25^b^± 0.127	<0.001	<0.001	0.005	<0.001	0.111	0.016	0.004
Bacteroidetes	AY244965_f	−	−0.77^a^± 0.269	0.12^b^± 0.203	0.42^b^± 0.164	<0.001	0.003	0.380	0.671	0.824	0.241	0.067
Bacteroidetes	BS11_f	−	−0.58^a^± 0.339	−0.24^ab^± 0.262	0.32^b^± 0.215	0.047	0.045	0.124	0.078	0.072	0.480	0.424
Bacteroidetes	GU304534_f	−	−0.52^a^± 0.500	−0.37^a^± 0.388	0.73^b^± 0.320	0.012	<0.001	0.274	0.321	0.949	0.671	0.622
Bacteroidetes	Prevotellaceae	−	0.18 ± 0.176	−0.18 ± 0.134	0.066 ± 0.110	0.161	<0.001	0.374	0.194	0.918	0.694	0.912
Bacteroidetes	RF16_f	−	−0.74^a^± 0.423	0.82^b^± 0.323	0.025^a^± 0.264	0.005	0.004	0.663	0.007	0.352	0.598	0.110
Bacteroidetes	S24-7_f	−	0.57 ± 0.365	−0.125 ± 0.281	−0.24 ± 0.231	0.102	<0.001	0.861	0.596	0.955	0.267	0.935
Chloroflexi	Anaerolinaceae	−	−1.20^a^± 0.378	0.46^b^± 0.288	0.12^b^± 0.234	<0.001	<0.001	0.594	0.118	0.135	0.052	0.153
Firmicutes	Acidaminococcaceae	+	−0.15 ± 0.400	−0.33 ± 0.307	−0.26 ± 0.253	0.918	<0.001	0.689	0.417	0.536	0.495	0.847
Firmicutes	Carnobacteriaceae	+	0.49 ± 0.347	0.40 ± 0.308	−0.044 ± 0.284	0.076	<0.001	0.211	0.038	0.008	0.008	0.072
Firmicutes	Christensenellaceae	−	−0.92^a^± 0.172	0.26^b^± 0.132	0.28^b^± 0.109	<0.001	<0.001	0.091	<0.001	0.102	0.040	0.070
Firmicutes	Erysipelotrichaceae	+	0.37^a^± 0.143	0.064^ab^± 0.110	−0.15^b^± 0.090	0.001	<0.001	0.735	0.051	0.341	0.365	0.260
Firmicutes	Eubacteriaceae	+	0.60 ± 0.352	0.061 ± 0.266	−0.28 ± 0.215	0.090	<0.001	0.009	0.134	0.995	0.423	0.016
Firmicutes	Lachnospiraceae	+	0.26^a^± 0.083	0.10^a^± 0.069	−0.20^b^± 0.061	<0.001	<0.001	0.131	<0.001	0.447	0.858	0.609
Firmicutes	Lactobacillaceae	+	0.25 ± 0.365	0.077 ± 0.302	−0.24 ± 0.262	0.298	<0.001	0.303	0.565	0.451	0.324	0.194
Firmicutes	Leuconostocaceae	+	−0.26 ± 0.516	0.20 ± 0.447	−0.18 ± 0.403	0.611	<0.001	0.130	0.936	0.173	0.099	0.104
Firmicutes	Ruminococcaceae	+	−0.30^a^± 0.079	-0.041^b^± 0.059	0.047^b^± 0.048	<0.001	<0.001	0.087	<0.001	0.045	0.132	0.248
Firmicutes	Streptococcaceae	+	0.24 ± 0.420	0.69 ± 0.339	−0.26 ± 0.289	0.053	<0.001	0.590	0.159	0.637	0.428	0.159
Firmicutes	Veillonellaceae	−	2.05^a^± 0.475	0.067^b^± 0.374	−0.85^c^± 0.312	<0.001	0.241	0.006	0.152	0.071	<0.001	0.096
Lentisphaerae	AF050559_f	−	−1.46^a^± 0.402	0.50^b^± 0.323	0.080^b^± 0.273	<0.001	0.029	0.386	0.017	0.993	0.090	0.110
Lentisphaerae	EF436358_f	−	−1.54^a^± 0.423	-0.60^a^± 0.333	1.02^b^± 0.278	<0.001	0.670	0.916	<0.001	0.084	0.539	0.861
Planctomycetes	Planctomycetaceae	−	−1.03^a^± 0.311	0.23^b^± 0.240	0.59^b^± 0.197	<0.001	0.001	0.748	0.017	0.989	0.539	0.668
Proteobacteria	Acetobacteraceae	−	0.054 ± 0.386	-0.13 ± 0.327	0.077 ± 0.290	0.704	<0.001	0.377	0.765	0.184	0.259	0.836
Proteobacteria	Gammaproteobacteria_c	−	−2.04^a^± 0.509	-0.66^b^± 0.394	0.97^c^± 0.325	<0.001	0.079	0.355	0.223	0.123	0.709	0.249
Proteobacteria	Paracaedibacteraceae	−	−0.68^a^± 0.330	-0.30^a^± 0.250	0.56^b^± 0.203	<0.001	0.718	0.208	0.989	0.117	0.792	0.004
Saccharibacteria	Saccharimonadaceae	+	−0.57^a^± 0.335	0.40^b^± 0.262	0.090^a^± 0.218	0.036	<0.001	0.086	0.006	0.093	0.020	0.061
Tenericutes	Acholeplasmataceae	−	−0.86^a^± 0.434	0.158^b^± 0.339	0.60^b^± 0.281	0.007	<0.001	0.262	0.991	0.359	0.249	0.845
Tenericutes	EF445272_f	−	−0.94^a^± 0.243	0.28^b^± 0.189	0.062^b^± 0.156	<0.001	<0.001	0.404	0.008	0.686	0.016	0.036

^a–c^Means within a row not sharing a common superscript letter differ significantly (p < 0.05).

^1^The analysis model included the fixed effects of group, region, and parity, and their interactions and the random effect of herd within region. The 29 families included are those that were considered as the 20 “influential” families with the best-fit for either group or region based on redundancy analyses. The phylum that each family belongs to and the Gram stain status are also included. The *n* was 257.

Primiparous cows had greater abundances of Anaerocella_f, Eubacteriaceae, and Veillonellaceae than multiparous cows (**Tables 3**, **4**). Region was significant for the abundance of all influential families except Veillonellaceae, EF436358_f, Gammaproteobacteria_c (tendency *p*= 0.079), and Paracaedibacteraceae. The cows from Canada had greater abundances of AY244965_f, GU304534_f, RF16_f, and Acetobacteraceae than cows from Australia and California. The cows from Canada also had greater abundances of BS11_f than cows from California, but Australian cows had similar abundances of BS11_f to cows from both California and Canada (**Tables 3**, **4**).

**Table 4:** The mean ± SE of the center-logged ratios of the relative abundances of ruminal bacterial families for each region and parity from a model that included the fixed effects of acidosis risk group, region, and parity and their interactions and the random effect of herd within region^1^.

Phyla	Family	Gram stain (+/-)	Region			Parity	
			Australia	California	Canada	Primiparous	Multiparous
Actinobacteria	Coriobacteriaceae	+	0.001^a^± 0.223	0.99^b^± 0.215	−0.78^c^± 0.228	0.13 ± 0.138	−0.11 ± 0.133
Bacteroidetes	Anaerocella_f	−	0.48^a^± 0.169	−0.35^b^± 0.161	0.045^b^± 0.182	0.14^a^± 0.111	−0.001^b^± 0.105
Bacteroidetes	AY244965_f	−	−0.019^a^± 0.219	−0.60^a^± 0.208	0.63^b^± 0.235	−0.019 ± 0.143	−0.051 ± 0.136
Bacteroidetes	BS11_f	−	−0.38^ab^± 0.300	−0.66^b^± 0.288	0.74^a^± 0.313	−0.055 ± 0.190	0.33 ± 0.182
Bacteroidetes	GU304534_f	−	−0.037^a^± 0.449	−1.39^a^± 0.432	1.50^b^± 0.466	−0.15 ± 0.283	0.10 ± 0.272
Bacteroidetes	Prevotellaceae	−	0.65^a^± 0.151	−1.14^b^± 0.144	0.42^a^± 0.159	−0.093 ± 0.097	0.079 ± 0.093
Bacteroidetes	RF16_f	−	−0.55^a^± 0.362	−0.69^a^± 0.346	1.14^b^± 0.382	−0.025 ± 0.232	0.24 ± 0.222
Bacteroidetes	S24-7_f	−	0.42^a^± 0.321	−1.68^b^± 0.308	−0.99^a^± 0.336	−0.061 ± 0.204	0.051 ± 0.195
Chloroflexi	Anaerolinaceae	−	−1.52^a^± 0.319	1.26^b^± 0.304	0.004^c^± 0.338	−0.006 ± 0.205	−0.27 ± 0.196
Firmicutes	Acidaminococcaceae	+	0.16^a^± 0.357	−1.85^b^± 0.343	0.66^a^± 0.370	−0.23 ± 0.224	−0.055 ± 0.216
Firmicutes	Carnobacteriaceae	+	−0.58^a^± 0.472	1.62^b^± 0.467	−0.20^a^± 0.445	0.100 ± 0.271	0.034 ± 0.268
Firmicutes	Christensenellaceae	−	−0.78^a^± 0.152	0.70^b^± 0.145	0.038^b^± 0.158	−0.077 ± 0.096	−0.075 ± 0.092
Firmicutes	Erysipelotrichaceae	+	0.65^a^± 0.124	0.091^b^± 0.119	−0.56^c^± 0.130	0.089 ± 0.118	−0.084 ± 0.114
Firmicutes	Eubacteriaceae	+	1.28^a^± 0.289	−0.46^b^± 0.274	−0.70^ab^± 0.309	0.16^a^± 0.188	−0.20^b^± 0.179
Firmicutes	Lachnospiraceae	+	−0.13^a^± 0.095	0.34^b^± 0.094	−0.16^a^± 0.093	0.050 ± 0.056	−0.001 ± 0.055
Firmicutes	Lactobacillaceae	+	−1.09^a^± 0.406	1.25^b^± 0.397	−0.084^a^± 0.398	−0.034 ± 0.241	−0.008 ± 0.236
Firmicutes	Leuconostocaceae	+	−1.85^a^± 0.656	1.79^b^± 0.648	0.001^a^± 0.626	−0.016 ± 0.381	−0.135 ± 0.376
Firmicutes	Ruminococcaceae	+	−0.23^a^± 0.065	0.19^b^± 0.062	−0.12^a^± 0.069	−0.030 ± 0.042	−0.033 ± 0.040
Firmicutes	Streptococcaceae	+	−0.66^a^± 0.434	2.05^b^± 0.422	−0.70^a^± 0.432	−0.084 ± 0.262	−0.020 ± 0.255
Firmicutes	Veillonellaceae	−	0.34 ± 0.451	−0.40 ± 0.436	0.34 ± 0.460	0.26^a^± 0.279	−0.066^b^± 0.269
Lentisphaerae	AF050559_f	−	0.091^a^± 0.408	−1.26^b^± 0.396	0.38^a^± 0.408	−0.16 ± 0.247	−0.22 ± 0.240
Lentisphaerae	EF436358_f	−	0.15 ± 0.402	−0.50 ± 0.388	0.10 ± 0.410	−0.075 ± 0.248	−0.072 ± 0.240
Planctomycetes	Planctomycetaceae	−	−0.65^a^± 0.276	0.65^b^± 0.265	0.27^b^± 0.288	0.051 ± 0.174	−0.059 ± 0.167
Proteobacteria	Acetobacteraceae	−	−1.33^a^± 0.461	−0.49^a^± 0.454	1.70^b^± 0.445	0.081 ± 0.270	−0.12 ± 0.266
Proteobacteria	Gammaproteobacteria_c	−	−0.87 ± 0.458	−0.80 ± 0.440	0.82 ± 0.475	−0.19 ± 0.288	0.073 ± 0.277
Proteobacteria	Paracaedibacteraceae	−	0.080 ± 0.274	−0.20 ± 0.261	0.061 ± 0.292	−0.024 ± 0.178	0.055 ± 0.169
Saccharibacteria	Saccharimonadaceae	+	−0.92^a^± 0.312	0.93^b^± 0.300	0.064^c^± 0.320	−0.12 ± 0.194	−0.047 ± 0.187
Tenericutes	Acholeplasmataceae	−	0.43^a^± 0.401	−1.28^b^± 0.386	0.94^a^± 0.412	−0.037 ± 0.250	0.290 ± 0.241
Tenericutes	EF445272_f	−	−0.75^a^± 0.221	0.44^b^± 0.213	−0.047^ab^± 0.229	0.030 ± 0.139	−0.14 ± 0.133

^a−c^Means within a row not sharing a common superscript letter differ significantly (p < 0.05).

^1^The 29 families included are those that were considered the 20 “influential” families with the best-fit for either group or region based on redundancy analyses. The phylum that each family belongs to and the Gram strain status are also included. The *n* was 257.

The cows from Australia had greater abundances of Anaerocella_f and lower abundances of Christensenellaceae and Planctomycetaceae than cows from other regions. Cows from Australia had a lower abundance of EF445272_f and greater abundance of Eubacteriaceae than cows from California, but their abundances of these families were similar to those in cows from Canada, and cows from California and Canada also had similar abundances (**Tables 3**, **4**). Cows from Australia had a greater abundance of Erysipelotrichaceae than cows from California, which was higher than that in cows from Canada.

The cows from California had greater abundances of Carnobacteriaceae, Lachnospiraceae, Lactobacillaceae, Leuconostocaceae, and Streptococcaceae and lower abundances of Prevotellaceae, S24-7_f, Acidaminococcaceae, AF050559_f, and Acholeplasmataceae, than those from other regions. The cows from California also had greater abundances of Coriobacteriaceae, Anaerolinaceae, Ruminococcaceae, and Saccharimonadaceae than cows from Australia, which was greater than abundances in cows from Canada (**Tables 3**, **4**).

**Figure 2:**
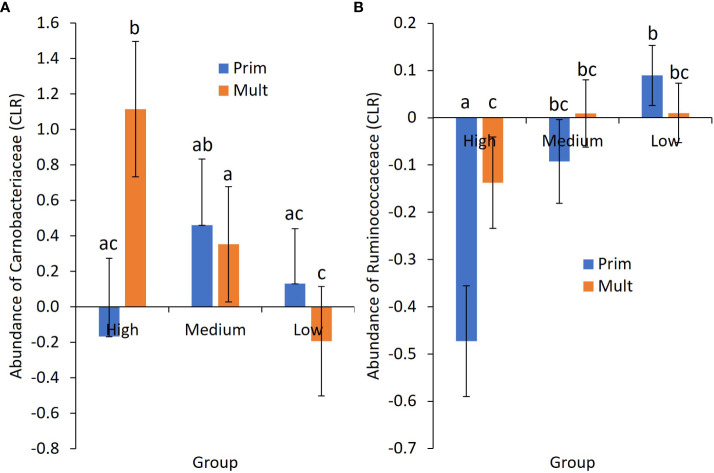
Mean ± SE center-logged ratio (CLR) of abundance for **(A)** the family Carnobacteriaceae and **(B)** the family Ruminococcaceae by acidosis risk group and parity. Prim, primiparous cows; Mult, multiparous cows. a–c Means not sharing a common superscript letter differ significantly (p < 0.05).

Group by region was significant for over one-third of the influential phyla (**Table 3**). Group by parity was significant for Carnobacteriaceae and Ruminococcaceae (**Figures 2A, B**). Region by parity was significant for Anaerocella_f, Carnobacteriaceae, Christensenellaceae, Veillonellaceae, Saccharimonadaceae, and EF445272_f. There were three-way interactions for Anaerocella_f, Eubacteriaceae, Paracaedibacteraceae, and EF445272_f, despite only a significant main effect for acidosis group and no significant two-way interactions.

### 3.2 Beta diversity—redundance analysis correlation biplot

**Figure 3:**
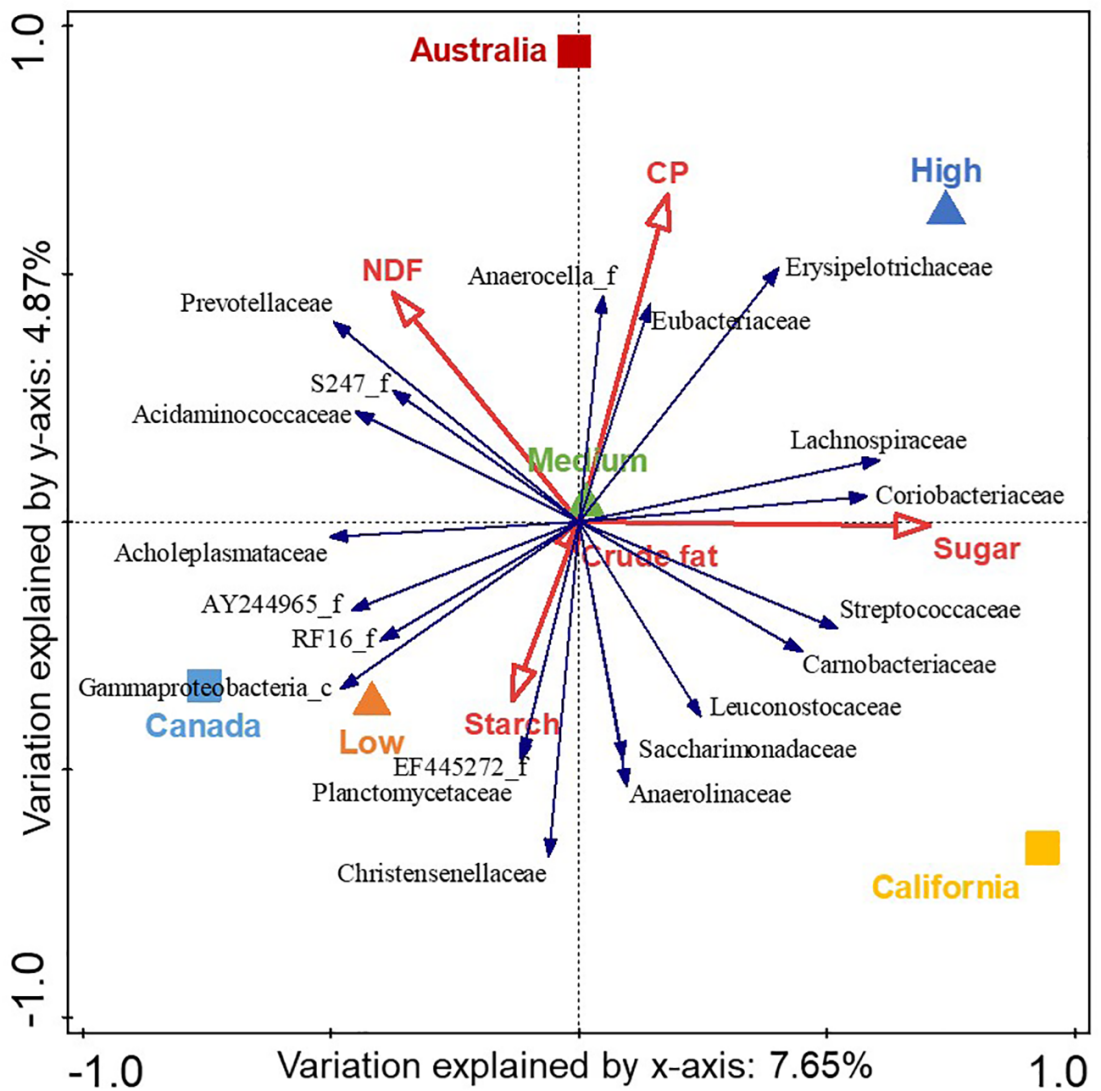
Correlation biplot of the redundancy analysis of the center-log-transformed relative abundance of bacterial families (blue arrows) with respect to acidosis risk group (triangles), region (squares), and nutrient explanatory variables (red arrows). The triangle or square is the midpoint of the samples in that group, whereas the red arrows indicate the direction in which the variable increases. The 20 bacterial families with the best fit to the variables are displayed where the length of the arrows are approximate correlation coefficients between the variables and acidosis risk and region, with relative abundance increasing in the direction of the arrow. The total variation associated with the variables is 17.6% and the eigenvalues for the first two axes are 0.08 and 0.05. The high- and low-risk samples were both different from the rest of the samples: high-risk samples were associated with 4.1% of the variation (p = 0.022), medium-risk samples with 0.7% of the variation (p = 0.154), and low-risk samples with 3.3% of the variation (p = 0.022). Each region was statistically different: California was associated with 6.3% of the variation (p = 0.022), Canada with 5.1% of the variation (p = 0.022), and Australia with 4.0% of the variation (p = 0.022). Sugar, crude protein (CP), neutral detergent fiber (NDF), and starch were all statistically different (p = 0.022), associated with 4.4%, 2.8%, 2.4%, and 1.0% of the variation, respectively. Crude fat was associated with 0.8% of the variation (p = 0.176).

**Figure 3** shows a correlation biplot of the redundancy analysis of bacterial families with respect to acidosis risk group, region, and dietary nutrients (namely, NDF, CP, sugar, starch, and crude fat). Only the 20 families with the best fit to this model are displayed and the *x* and *y* coordinates and Cfit2 values for each of these bacterial families are shown in **Supplementary Table 1**. The variables explained 17.6% of the total variation. The high- and low-risk samples both differed to the rest of the samples and presented in inverse quadrants to each other; the high-risk acidotic category was associated with 4.1% of the variation (*p*= 0.022), the medium-risk category was associated with 0.7% of the variation (*p*= 0.154), and the low-risk category was associated with 3.3% of the variation (*p*= 0.022) and presented in the center of the biplot. Each region was statistically different and presented in different quadrants; California was associated with 6.3% of the variation (*p*= 0.022), Canada was associated with 5.1% of the variation (*p*= 0.022), and Australia was associated with 4.0% of the variation (*p*= 0.022). Sugar, CP, NDF, and starch were all statistically different (*p*= 0.022) and associated with 4.4%, 2.8%, 2.4%, and 1.0% of the variation, respectively. Crude fat was associated with 0.8% of the variation (*p*= 0.176). Sugar and CP content were associated with the high-risk group, whereas starch was associated with the low-risk group. The abundance of the family Erysipelotrichaceae was that most associated with the high-risk group; that of the family Gammaproteobacteria_c was most associated with the low-risk group; that of the family Prevotellaceae was most associated with NDF; and those of Coriobacteriaceae, Lachnospiraceae, and Streptococcaceae were most associated with sugar.

### 3.3 Bacterial predictors of groups

#### 3.3.1 Phyla level

There were five predictive phyla for both the high- and low-risk acidosis groups and none for the medium-risk group (**Table 5**), with the referent group comprising all cattle that were not in the respective group. Of these, four phyla were common to both groups (Firmicutes, Lentisphaerae, Planctomycetes, and Tenericutes), but with opposite point directions. The odds of a cow being in the high-risk group compared with the other groups was associated with an approximately 7.5-times greater abundance of Firmicutes; an approximately 1.3-times greater abundance of Spirochaetes; and reduced abundances of Lentisphaerae, Planctomycetes, and Tenericutes (**Table 5**). Similarly, the odds of being in the low-risk group were associated with a 2.3-times increase in the abundance of Tenericutes, and a 1.4-times increase in the abundances of Planctomycetes and Lentisphaerae. The odds of being in the low-risk group were associated with a decreased odds of abundance of Fibrobacteres by 0.74, and of Firmicutes by 0.062 compared with cattle not in the low-risk group. For the medium-risk group, only Fibrobacteres was significant in the univariable analysis and was not significant in the backward stepwise regression model that included the fixed effects of herd within region.

**Table 5:** The Gram stain status, odds ratio (OR), SE, 95% CI, and significance of ruminal bacterial phyla that had significant (*p*< 0.05) center-logged ratios of relative abundance within the high- and low-risk groups in respectively backwards elimination regression models that included the fixed effects of herd nested within region^1^.

Phylum	Gram stain (+/−)	OR	SE	95% CI	*p*-value	AUC
High-risk group^2^						0.925
Firmicutes	+	7.469	7.435	1.062 to 52.545	0.043	
Spirochaetes	−	1.278	0.138	1.034 to 1.579	0.023	
Lentisphaerae	−	0.704	0.065	0.589 to 0.843	<0.001	
Planctomycetes	−	0.696	0.086	0.546 to 0.887	0.003	
Tenericutes	−	0.439	0.150	0.225 to 0.857	0.016	
Low-risk group^3^						0.930
Tenericutes	−	2.344	0.845	1.157 to 4.750	0.018	
Planctomycetes	−	1.409	0.216	1.043 to 1.901	0.025	
Lentisphaerae	−	1.337	0.133	1.100 to 1.626	0.004	
Fibrobacteres	−	0.741	0.076	0.606 to 0.907	0.004	
Firmicutes	+	0.062	0.057	0.010 to 0.373	0.002	

AUC, area under the curve of the model.

^1^There were no significant bacterial phyla from the medium-risk group.

^2^The referent group comprises all non-high-risk cows (i.e., cows from the medium- and low-risk groups).

^3^The referent group comprises all non-low-risk cows (i.e., cows from the high- and medium-risk groups).

#### 3.3.2 Family level

The high-risk group had the most associated families (*n*= 9), followed by the low-risk group (*n*= 7), and the medium-risk group (*n*= 5) (**Table 6**), with the referent group comprising all cattle that were not in the respective group. There was a modest amount of overlap in associated families, with four families (Lachnospiraceae, Planctomycetaceae, Gammaproteobacteria_c, and Peptostreptococcaceae) being associated with more than one group, all with opposite point directions. The greatest change in point direction was for Lachnospiraceae between the high- and low-risk groups, followed by Planctomycetaceae and Gammaproteobacteria_c. Peptostreptococcaceae was associated with both the medium- and low-risk groups.

**Table 6:** The odds ratios (OR), SE, 95% CI, and significance of ruminal bacterial families that had significant (*p*< 0.05) center-logged ratios of relative abundance within acidosis risk groups in backwards stepwise elimination models that included the fixed effects of herd nested within region.

Family	Phylum	Gram stain (+/-)	OR	SE	95% CI	*p*-value	AUC
High-risk group^1^							0.958
Lachnospiraceae	Firmicutes	Gram positive	8.854	6.572	2.067 to 37.925	0.003	
Coriobacteriaceae	Actinobacteria	Gram positive	2.245	0.744	1.173 to 4.299	0.015	
Chthoniobacteraceae	Verrucomicrobia	Gram negative	1.881	0.502	1.115 to 3.173	0.018	
Spirochaetaceae	Spirochaetes	Gram negative	1.361	0.161	1.079 to 1.717	0.009	
Lactobacillaceae	Firmicutes	Gram positive	1.336	0.197	1.000 to 1.784	0.050	
Planctomycetaceae	Planctomycetes	Gram negative	0.668	0.110	0.484 to 0.922	0.014	
Gammaproteobacteria_c	Proteobacteria	Gram negative	0.558	0.106	0.384 to 0.810	0.002	
FN377789_o	Verrucomicrobia	Gram negative	0.382	0.148	0.179 to 0.816	0.013	
Mogibacterium_f	Firmicutes	Gram positive	0.263	0.105	0.121 to 0.573	0.001	
Medium-risk group^2^							
Exiguobacteriaceae	Firmicutes	Gram positive	2.449	0.779	1.313 to 4.569	0.005	0.926
Peptostreptococcaceae	Firmicutes	Gram positive	1.614	0.270	1.163 to 2.239	0.004	
Streptococcaceae	Firmicutes	Gram positive	1.424	0.156	1.150 to 1.764	0.001	
RF16_f	Bacteroidetes	Gram negative	1.395	0.145	1.138 to 1.709	0.001	
Alcaligenaceae	Bacteroidetes	Gram negative	0.628	0.125	0.426 to 0.927	0.019	
Low-risk group^3^							0.953
Planctomycetaceae	Planctomycetes	Gram negative	1.702	0.292	1.215 to 2.383	0.002	
Gammaproteobacteria_c	Proteobacteria	Gram negative	1.363	0.128	1.134 to 1.639	0.001	
Bacteroidales_o	Bacteroidetes	Gram negative	0.786	0.086	0.634 to 0.973	0.027	
Carnobacteriaceae	Firmicutes	Gram positive	0.735	0.097	0.568 to 0.952	0.020	
Fibrobacteraceae	Fibrobacteres	Gram negative	0.730	0.077	0.594 to 0.898	0.003	
Peptostreptococcaceae	Firmicutes	Gram positive	0.713	0.118	0.515 to 0.987	0.042	
Lachnospiraceae	Firmicutes	Gram positive	0.100	0.065	0.028 to 0.361	<0.001	

AUC, area under the curve of the model.

^1^The referent group comprises all non-high-risk cows (i.e., cows from the medium- and low-risk groups).

^2^The referent group comprises all non-medium-risk cows (i.e., cows from the high- and low-risk groups).

^3^The referent group comprises all non-low-risk cows (i.e., cows from the high- and medium-risk groups).

Of the families associated with the high-risk acidosis group, approximately half were families that had increased abundance compared with cows not in this group, with abundances ranging from increased odds of approximately 8.9 times for the Lachnospiraceae family to increases of 1.34 times for the Lactobacillaceae family (**Table 6**). The decreased odds for this group ranged from 0.67 for the Planctomycetaceae family through to 0.26 for Mogibacterium_f. For the low-risk group, just over two-thirds of the associated families were those that decreased in abundance, ranging from Bacteroidales_o, which decreased by odds of 0.79, to Lachnospiraceae, which decreased by odds of 0.10. The increased odds for the low-risk group were more modest than the other groups, with odds of 1.7 times for Planctomycetaceae and odds of 1.4 times for Gammaproteobacteria_c. All families that were associated with the medium-risk group all increased in abundance, apart from Alcaligenaceae. The greatest increase was for the Exiguobacteriaceae family, at approximately 2.5 times. There was an increase in Streptococcaceae abundance by approximately 1.4 times for this group association.

### 3.4 Dietary nutrient predictors of groups

#### 3.4.1 Phyla level

The results from the five most abundant phyla and those that were associated with the group in backwards stepwise models totalled nine phyla of interest and are reported in **Table 7**. The results from the 26 phyla that had at least one sample with an RA of > 0.3%, which include the nine phyla of interest, are in **Supplementary Table 2**. All phyla of interest had at least one nutrient that was a significant predictor of abundance. The abundance of Actinobacteria was the only phyla with which all 5 nutrients were associated. Fibrobacteres and Planctomycetes were only associated with one predictive nutrient. Of the nine phyla of interest, sugar was the most commonly predictive nutrient and was significant in seven of the models (78%), followed by CP in five (56%), and crude fat in four (44%). Although abundances of Actinobacteria and Firmicutes were positively associated with dietary nutrients, all other phyla were negatively associated with dietary nutrients. Statistical confounding occurred with NDF and sugar for Bacteroidetes, crude fat and sugar for Firmicutes, and starch and crude fat for Spirochaetes. In the predictive models of all the 26 phyla with at least one sample with an RA of > 0.3% that included the phyla of interest, CP predicted 50.0%, sugar predicted 38.5%, NDF predicted 26.9%, starch predicted 30.8%, and crude fat predicted 38.5% of these phyla (**Supplementary Table 2**). This indicates that there was a similar pattern of response to dietary nutrients that was relatively consistent regardless of abundance.

**Table 7:** The coefficient, SE, and significance for diet nutrient components from a backwards stepwise elimination regression, that included the fixed effect of region for the five phyla with the greatest relative abundances and the phyla that were significant predictors of acidosis risk group^1^.

Phylum	Coefficient	SE	*p*-value	RSME	Mean abundance		Reason included
					Relative (%)	CLR	
Actinobacteria				0.942	8.69	−0.014	HA
CP	0.114	0.064	0.077				
Sugar	0.146	0.080	0.068				
NDF	0.049	0.028	0.084				
Starch	0.089	0.043	0.040				
Crude fat	0.237	0.120	0.048				
Bacteroidetes				0.697	28.6	0.000	HA
Sugar	−0.085	0.048	0.080				
NDF	−0.009	0.013	0.480				
Fibrobacteres				1.185	0.06	−0.023	LOW
Sugar	−0.157	0.088	0.075				
Firmicutes				0.198	50.5	0.000	HA, HIGH, LOW
Sugar	0.028	0.015	0.068				
Crude fat	0.023	0.016	0.163				
Lentisphaerae				1.505	0.61	−0.007	HIGH, LOW
CP	−0.166	0.096	0.083				
Sugar	−0.288	0.108	0.008				
Planctomycetes				1.150	0.67	−0.019	HIGH, LOW
CP	−0.214	0.074	0.004				
Proteobacteria				0.569	3.61	0.017	HA
CP	−0.068	0.036	0.058				
Sugar	−0.168	0.042	<0.001				
NDF	−0.034	0.014	0.013				
Spirochaetes				1.603	1.41	0.018	HIGH
Sugar	−0.364	0.130	0.005				
Starch	−0.098	0.061	0.112				
Crude fat	−0.445	0.208	0.032				
Tenericutes				0.332	4.62	0.016	HA, HIGH, LOW
CP	−0.099	0.021	<0.001				
Crude fat	−0.067	0.033	0.040				

CP, crude protein; NDF, neutral detergent fiber; HA, high abundance; HIGH, significant predictor for classification into the high-risk acidosis group; LOW, significant predictor for classification into the low-risk group for acidosis. ^1^Nutrients with a significance of *p*< 0.100 remained in the model, unless there was confounding. The root mean square error (RMSE) for each model (phyla), abundance [relative and center-logged ratio (CLR)] for each phylum and the reason for inclusion are given.

#### 3.4.2 Family level

All the influential families, except for Anaerolinaceae, Lactobacillaceae, and Leuconostocaceae had at least one diet nutrient that predicted their abundance (**Table 8**). The model for the Coriobacteriaceae family contained all five nutrients. The nutrients that were most predictive of the families were CP and sugar, both being significant at *p*< 0.100 in 52% of the 29 influential families, followed in descending order of prediction by crude fat at 34%, NDF at 24%, and starch at 7%. Confounding occurred for EF445272_f with starch and crude fat (**Table 8**).

**Table 8:** The coefficient, SE, and significance for diet nutrient components from a backwards stepwise elimination regression that included the fixed effect of region, for a combination of the 20 most influential families with the best-fit in redundancy analysis biplots for acidosis risk group and region, totaling 29 families^1^.

Family	Coefficient	SE	*p*-value	RMSE	Abundance		Rank	
					Relative (%)	CLR	Group	Region
Acetobacteraceae				1.489	0.10	0.006	19	4
CP	−0.168	0.097	0.084					
Sugar	−0.431	0.113	<0.001					
Acholeplasmataceae				1.699	0.16	-0.006	11	18
Crude fat	−0.214	0.129	0.097					
Acidaminococcaceae				1.317	2.56	0.023	−	14
Sugar	−0.251	0.100	0.012					
AF050559_f				1.551	0.14	0.010	6	−
Sugar	−0.323	0.112	0.004					
Anaerocella_f				0.666	0.011	0.001	15	−
Crude fat	0.106	0.044	0.016					
Anaerolinaceae		None			0.48	−0.010	−	7
AY244965_f				0.996	0.94	−0.003	8	20
CP	−0.161	0.059	0.006					
NDF	−0.037	0.021	0.074					
BS11_f				1.307	0.031	0.004	18	17
Crude fat	−0.186	0.090	0.039					
Carnobacteriaceae				1.657	0.19	−0.017	−	9
Sugar	0.252	0.134	0.060					
Christensenellaceae				0.733	4.78	−0.003	5	6
CP	−0.178	0.045	<0.001					
Crude fat	−0.101	0.061	0.099					
Coriobacteriaceae				0.911	2.29	−0.005	13	3
CP	0.168	0.054	0.002					
Sugar	0.128	0.067	0.057					
NDF	0.058	0.024	0.015					
Starch	0.062	0.037	0.092					
Crude fat	0.184	0.099	0.062					
EF436358_f				1.454	0.42	0.001	4	−
CP	−0.158	0.093	0.087					
Sugar	−0.240	0.104	0.022					
EF445272_f				0.770	0.60	−0.003	12	−
CP	−0.189	0.051	<0.001					
Starch	−0.033	0.030	0.275					
Crude fat	−0.193	0.097	0.047					
Erysipelotrichaceae				0.538	3.84	−0.001	3	2
CP	0.095	0.035	0.006					
Sugar	0.118	0.044	0.007					
NDF	0.027	0.013	0.029					
Eubacteriaceae				1.035	0.064	−0.007	20	10
CP	0.242	0.065	<0.001					
Sugar	0.258	0.076	0.001					
NDF	0.098	0.025	<0.001					
Gammaproteobacteria_c				1.622	0.84	0.010	2	13
CP	−0.309	0.106	0.004					
Sugar	−0.532	0.128	<0.001					
NDF	−0.116	0.040	0.004					
GU304534_f				1.971	0.60	−0.007	10	15
NDF	−0.084	0.039	0.030					
Lachnospiraceae				0.329	15.4	−0.001	1	16
CP	0.051	0.021	0.016					
Sugar	0.065	0.027	0.016					
Crude fat	0.097	0.030	0.001					
Lactobacillaceae		None			2.06	−0.013	−	14
Leuconostocaceae		None			0.58	−0.018	−	12
Paracaedibacteraceae				1.019	0.041	0.003	16	
Sugar	−0.108	0.074	0.142					
Planctomycetaceae				1.150	0.67	−0.011	7	−
CP	−0.214	0.074	0.004					
Prevotellaceae				0.757	18.8	0.004	−	1
Sugar	−0.095	0.046	0.037					
RF16_f				1.377	0.61	0.005	14	−
CP	−0.235	0.090	0.009					
Sugar	−0.292	0.110	0.008					
Crude fat	−0.290	0.135	0.031					
Ruminococcaceae				0.268	17.3	−0.001	17	−
CP	−0.035	0.018	0.049					
S24-7_f				1.27	2.46	0.0059	−	11
Sugar	−0.117	0.098	0.230					
Saccharimonadaceae				0.963	0.16	−0.002	−	19
CP	−0.255	0.060	<0.001					
Crude fat	−0.149	0.082	0.071					
Streptococcaceae				1.604	0.35	−0.014	−	5
Sugar	0.382	0.129	0.003					
Veillonellaceae				1.514	0.37	0.009	9	−
CP	0.180	0.095	0.060					
NDF	0.073	0.036	0.042					
Crude fat	0.309	0.149	0.039					

CP, crude protein; NDF, neutral detergent fiber. ^1^Nutrients with a significance *p*< 0.100 remained in the model, unless there was confounding. The root mean square error (RMSE) for each model (family) is given, the abundance [relative and center-logged ratio (CLR)] for each family and its rank in the 20 most influential families with the best-fit for acidosis risk group and region are also provided.

## 4 Discussion

This study intended to identify the ruminal bacterial taxa associated with ruminal acidosis and determine if the abundance of bacterial taxa could be predicted based on the intake of diet nutrient components. The study is part of a series of studies (Golder et al., 2023b; Golder et al., 2023c) designed to improve our understanding of acidosis risk status and, ultimately, the control of ruminal acidosis. Ruminal acidosis in dairy cattle, as opposed to clinical acidosis, is a subclinical or “subacute” condition (Nagaraja and Titgemeyer, 2007; Plaizier et al., 2018; Plaizier et al., 2022); hence, changes in the physiology may be relatively subtle and large studies are required to evaluate such changes (Bramley et al., 2008; O’Grady et al., 2008). Improving understanding of the complexity of the rumen community, let alone a multifactorial disorder such as ruminal acidosis, requires multiple omics approaches (Gruninger et al., 2019). Metataxonomic studies alone are limited in that variation can occur between sampling, sample storage, and sequencing methods, bioinformatic pipelines, and databases; they provide little information on function, and taxonomy assignment differs with the variable region sequenced of the 16S rRNA gene (Pollock et al., 2018; Gruninger et al., 2019). In this study, we integrate metataxonomic data with phenotypic observations of dietary exposure and rumen function to provide greater insight. Although the herds were purposively selected for their willingness to participate, the multi- herd and multi-country study design suggests that results should be widely applicable.

Of the 293 early-lactation Holstein cattle from four geographical regions that were classified into three acidosis risk groups (high, medium, and low) by Golder et al. (2023b) using the model by Bramley et al. (2008), only 261 cows from three regions were suitable for mixed-model analysis. Of these, 26.1% of the cows were classified in the high-risk group, 26.8% in the medium-risk group, and 47.1% in the low-risk group (Golder et al., 2023b). The high-risk group had rumen and production characteristics consistent with a model of acidosis that reflected a rapid rate of fermentation, the medium-risk group contained cows that may be inappetant, had not eaten recently or were in recovery from acidosis, and it was proposed that the low-risk group would contain cattle that were well fed with a slower rumen fermentation of carbohydrates (Golder et al., 2023b).

### 4.1 Prediction of acidosis group by bacterial taxa

The relatively few phyla (only five in total and four overlapping between acidosis risk groups) and families associated with acidosis and ruminal conditions support the theory that ruminants have a core microbiota (Jami and Mizrahi, 2012). In a large cohort study where dairy cattle had the same diet and management, core genera accounted for 53.9% of correlations in a correlation network analysis among rumen bacteria, rumen short-chain fatty acids, and lactation performance (Xue et al., 2018). Large inter-animal variation in the relative abundance of core taxa was also observed (Xue et al., 2018). The large overlap in the number of influential families between acidosis group and region in the redundancy analyses adds further support for the existence of a core microbiota and emphasizes the dynamic nature of the non-core microbiota. The opposing point direction for coefficients for the phyla and families that predicted the high- and low-risk groups further supports a hypothesis that these are key core taxa that shift during ruminal disturbance. Furthermore, the consistency of these observations supports our use of the backward stepwise statistical approach to provide meaningful outcomes. Xue et al. (2018) suggest that enhanced knowledge of the core and non-core rumen microbiota enables their manipulation, and we concur with this view.

Golder et al. (2023b) found that the Firmicutes were the most abundant phylum for all groups and were most abundant in the high-risk acidosis group. The Firmicutes, Bacteroidetes, and Proteobacteria phyla are usually considered the dominant phyla in cattle (Hook et al., 2011). The Lachnospiraceae were the most abundant family in the high-risk group. Thus, it is not surprising that the Firmicutes phylum and the Lachnospiraceae family had the greatest increase in abundance in the models for the high-risk group, and that the inverse was observed for the low-risk group. The phylum with the second highest RA for all groups, Bacteroidetes (Golder et al., 2023b), was not associated with acidosis risk in our models. Bacteroidetes often have an inverse association with the RA of Firmicutes. Thus, taxa that had an increased likelihood of being increased in abundance in the low-risk group were phyla that were present in a low abundance. These findings align with the premise of Nagaraja and Titgemeyer (2007) that gram-positive Firmicutes may displace the gram-negative Bacteriodetes during ruminal acidosis. This also likely explains the absence of the abundant family Prevotellaceae, which is a member of the Bacteroidetes phylum, from the final models. The Prevotellaceae family contains a diverse population of species (Krieg, 2010), which may limit its value in prediction. Interestingly, Ruminococcaceae, a member of the Firmicutes phylum, was the third most abundant bacterial family overall on a RA basis and was the family with the greatest RA in the low-risk group that was also not associated with group inclusion, perhaps for a similar reason.

It is logical that the class Gammaproteobacteria from the Proteobacteria phylum was associated with groups as it contains a range of diverse medically important gram-negative rod bacteria, including *Escherichia coli, Salmonella* spp., and *Klebsiella* spp. from the Enterobacteriaceae family; *Pasteurella* spp. from the Pasteurellaceae family; *Yersinia pestis* (bubonic plague) from the Yersiniaceae family; and *Vibrio* spp. from the Vibrionaceae family.

### 4.2 High-risk group

This group was characterized by increased abundances of Anaerocella_f [which do not utilize carbohydrates (Abe et al., 2012)] and Veillonellaceae [which has various characteristics that include lactate utilization (Hungate, 1966; Bergey et al., 2011)], which suggests a higher risk of acidosis. Decreased abundances of several bacterial families with diverse characteristics (AF050559_f, Anaerolinaceae, AY244965_f, Christensenellaceae, EF445272_f, Gammaproteobacteria_c, Planctomycetaceae, and Ruminococcaceae) were associated with an increased risk of acidosis. These taxa belong to an array of different phyla that were not consistent in Gram stain and of these, only Gammaproteobacteria_c and Planctomycetaceae were also in the predictive model. The increased odds in the predictive model of the abundance of Lactobacillaceae, which is a family of lactic acid producers (Bergey et al., 2011), supports the classification of this group as high risk (Golder et al., 2023b). A high level of lactic acid accumulation in the rumen is most likely responsible for clinical acidosis only (Nagaraja and Titgemeyer, 2007). Clinical acidosis is less prevalent than milder forms of acidosis. Lactate concentrations concentrations did not differ among the acidosis risk groups in our study (Golder et al., 2023b); hence, our ruminal bacterial community composition should reflect this. This study was not an acidosis challenge study; thus, we were not likely to observe a high prevalence of clinical acidosis in the study population. Our data, being broader and from a range of herds with differing management and diets, is not directly comparable to evaluations of rumen bacterial community compositions from acidosis challenge studies that induce acidosis with particular substrates. Golder et al. (2012b) found that lactate concentrations peaked within 20 minutes of feed consumption but declined rapidly. Our rumen samples were not collected immediately after feeding, and it is possible that if lactate was generated then it had already been metabolized, with portions converted into propionate, valerate, and caproate, which are synthesized from three lactate molecules (Annison and Lewis, 1959), as these fermentation products were also highest in the high-risk acidosis group. Thus, perhaps there was a population of lactate utilizers present in this group. This suggestion is further supported by an increased abundance of the Veillonellaceae family in this group, which contains *Megasphaera elsdenii*, one of the producers of valerate from lactate (Hungate, 1966; Stewart et al., 1997).

The traditional model of aetiology of ruminal acidosis, which is largely based on culture-based techniques, suggests that *Streptococcus bovis* and lactic acid utilizers are primarily responsible for acidosis (Nocek, 1997). Culture-based techniques are widely recognized to have limitations in the evaluation of microbial populations, namely their vast underestimation of microbial diversity and richness (McSweeney et al., 2007; Fernando et al., 2010). There is an increasing body of evidence showing that acidosis is complex and that this model of the aetiology of ruminal acidosis needs refinement (Britton and Stock, 1989; Plaizier et al., 2018; Plaizier et al., 2022).

Our work shows that several bacterial taxa are likely to be involved in the etiology of ruminal acidosis. This is consistent with the knowledge that the rumen microbiome is dynamic with diversified adaptive functions (Gharechahi et al., 2021). The microbiome has high metabolic redundancy (Taxis et al., 2015), which probably explains why multiple microbial taxa may be associated with acidosis. Rumen microbes have symbiotic relationships with each other (Hungate, 1966); however, a survey by Henderson et al. (2015) of several different ruminants from different parts of the globe that are likely to have differed vastly in diet and management found that rumen bacteria rarely had similar co-occurrence patterns. It is critical to consider the difference between abundance compared to co-dependency compared to function. Metabolically active bacteria within and without biofilms may facilitate the growth and multiplication of other bacteria through substrate influence in the exometabolome (Morris et al., 2012; Douglas, 2020). Our study suggests there is enough commonality in the microbiome between acidosis cases in early-lactation Holsteins to use the abundance of rumen bacteria to predict acidosis risk. Currently, collection, storage, processing, interpretation of results, costs, and turn-around time of results prevent this from being a viable method of diagnosis for producers. Development of a rapid cow side test, used in combination with other diagnostic methods and clinical history, may be viable in the future.

### 4.3 Medium-risk group

Overall, the medium-risk group had very few associations with the microbiome, only being characterized by increased abundances of RF16_f and Saccharimonadaceae. These Gram-negative and Gram-positive bacterial families, respectively, although knowledge on them and their substrates is limited, are frequently found in cattle (Abbas et al., 2020; Daghio et al., 2021; Ouyang et al., 2021). The medium-risk group did not have any predictive phyla and had the smallest number of predictive families. This is consistent with the lack of differences in milk production (Golder et al., 2023b). However, Bainbridge et al. (2016) suggested that correlations between milk production and rumen microbial populations do not imply causation, as both may be strongly influenced by multiple factors. This group did have distinctive ruminal metabolomic measures, having the greatest rumen pH and least acetate, propionate, butyrate, valerate, iso-butyrate, iso-valerate, and caproate concentrations, and numerically lowest total VFA (Golder et al., 2023b). It is evident from these measures and the PCA plots that cattle in this group differ from those of the other two groups, but we may not have captured all the sources of heterogeneity. Golder et al. (2023b) suggested that this group could contain underfed cows, cows that are inappetent or that were not recently feeding, or cows in recovery from acidosis or an illness, as the mean total VFA was low. The concentration of ruminal VFA is directly related to bacterial community composition, amount of fermentation, and rumen epithelial absorption (Brockman, 2005). The greater abundance of Streptococcoceae in this group is interesting. This family contains *Streptococcus bovis*, which has traditionally been associated with the etiology of acidosis by precipitating a decline in rumen pH and consequent increase in lactic acid (Nocek, 1997). Interestingly, in grain-based subacute ruminal acidosis studies, responses have differed with both increases (Fernando et al., 2010) and decreases (Petri et al., 2013; Plaizier et al., 2017) observed in the populations of *S. bovis.* Neither lactic acid nor the abundance of Lactobacillaceae, the family that includes of the *Lactobacillus* genus, were increased in this group. Three out of the five predictive taxa were not included in the 29 influential taxa and were present in only minor abundances, but the three with the highest odds were all Gram-positive members of the Firmicutes phylum, supporting the hypothesis that this group could be recovering from acidosis. An increase in Firmicutes numbers occurs with an increase in rapidly fermentable carbohydrates (Hungate, 1966; Mao et al., 2013; Plaizier et al., 2022). Given the prevalence of cattle categorized as high risk for acidosis in the study population, and the time that is likely needed for the rumen to recover from dysbiosis, a population of cattle should exist that is in recovery from acidosis, and perhaps the medium-risk group represents these. Brede et al. (2020) showed that the rumen bacterial abundance of most bacterial groups returned to initial abundance 5 days after a subacute ruminal acidosis challenge using a rumen simulation technique (RUSITEC).

### 4.4 Low-risk group

The low-risk group was characterized by increased abundances of Gammaproteobacteria_c, EF436358_f, GU304534_f, and Paracaedibacteraceae, and decreased abundances of Lachnospiraceae and Veillonellaceae. Many members of the Gammaproteobacteria class have deleterious effects in cattle but are also natural inhabitants of the rumen. The release of LPS from the lysis of Gram-negative bacteria such as these may contribute to the pathogenesis of ruminal acidosis (Plaizier et al., 2018). The fact that these were more abundant in the low-risk group than in the high-risk group suggests that cattle in the low-risk group were at risk of disorders other than acidosis, that these bacteria may not pose a concern, or that these cattle had more robust and resilient microbial ecosystems that resist the potential negative impacts of these bacteria. It could be expected that some cattle would be in a state of flux between acidosis risk groups; each cow has a different distance to the centroid of each acidosis risk group, which reflects the strength of association at the time of sampling (Golder et al., 2023b). Ruminal acidosis is a multifactorial disorder and has substantial sequelae (Enemark, 2008; Plaizier et al., 2008). There may be additional sequelae that are yet to be identified as clinically or metabolically associated with acidosis.

The Planctomycetaceae family, which had the largest increase in odds ratio in the models, is from the Planctomycetes phylum, which is Gram-negative and plays a role in global carbon and nitrogen cycles (Wiegand et al., 2018). Many species of the Planctomycetes phylum are involved with anaerobic ammonium oxidation, a function consistent with the low-risk group having the highest ammonia concentrations (Golder et al., 2023b); however, these are usually found in the Brocadiaceae family (Wiegand et al., 2018). It is interesting that a decrease in the abundance of Fibrobacteraceae, a group likely to contain fiber digesters, was associated with increased odds of being in the low-risk group when we had hypothesized that the opposite would occur. Krajcarski-Hunt et al. (2002) showed that the rumen digestion of NDF from grass hay, legume hay, and corn silage was reduced by induced subacute ruminal acidosis.

Golder et al. (2023b) and Bramley et al. (2008) proposed that cattle in the low-risk group may have had a greater synergy between ruminal energy and protein metabolism than those in the high-risk group. This leads to slower fermentation, favoring the production of acetate and butyrate, which provide “safer” electron sinks and overall a more stable rumen. A lower abundance of Firmicutes at the phylum level, in addition to the family members of this phylum, the Carnobacteriaceae, Peptostreptococcaceae, and Lachnospiraceae, having increased odds of being in the low-risk group supports these cattle being at a lower risk of acidosis. The Carnobacteriaceae produce lactic acid as their major end product (Bergey et al., 2011), which is considered an “unsafe” electron sink and has a pKa of 3.0, compared with that of 4.8 for ruminal VFA (Oetzel, 2003). A decreased abundance of Veillonellaceae in the mixed-model analysis provides further support to this hypothesis. The Tenericutes phylum, which contains the *Mycoplasma* genus, had the greatest association with odds of inclusion in the low-risk group at the phylum level, but none of the predictive families for the low-risk group is a member of this phylum. The pattern of significance for the abundance of families was more similar between the medium- and low-risk groups than between the high- and low-risk groups.

### 4.5 Dietary nutrient predictors of bacterial abundance

Golder et al. (2023b) suggested that region may act to confound acidosis group determination for some measures in this study and hypothesized that this would result from differences in diet and management among regions. Although the multi-center, multi-country study design provides strong external validity of our findings, the regional effects and their influence on group associations may be a limitation. Bacteria utilize different substrates (Hungate, 1966), thus the presence and abundance of bacteria should be predicted from dietary substrates. The dietary nutrient components that were most consistently associated with our key bacterial taxa were sugars and CP.

Cattle can be exposed to a range of sugar contents from different sources in their diet. Sugars have a greater k_d_ than starch and other carbohydrate fractions (Sniffen et al., 1992) and are therefore more likely to overwhelm the buffering capacity of the rumen than less readily fermentable carbohydrates. Sugars are more likely to be fermented into lactic acid than starches (Harmon et al., 1985; Heldt et al., 1999; Golder et al., 2012b) and create an increased risk of ruminal acidosis (Nagaraja and Titgemeyer, 2007; Golder et al., 2012b; Golder et al., 2014a).

The potential involvement of dietary protein content in the pathogenesis of ruminal acidosis is largely unexplored. The supply of rumen-degradable protein has influenced organic acid and lactate pool sizes (Hall, 2013) and increases in levels of rumen-available protein have increased organic acid concentrations, regardless of the levels of rumen-available carbohydrate (Herrera-Saldana and Huber, 1989). The rumen-undegradable portion of protein from the diet bypasses the rumen and is absorbed as amino acids in the small intestine, thereby supplying approximately 30% more energy than starch (Klopfenstein, 2001) without the generation of hydrogen in the rumen. A total of 8% of the dry weight of bacteria is hydrogen (Todar, 2012), which provides a considerable sink for the hydrogen generated during ruminal catabolism of carbohydrates. Consequently, it appears possible that protein can be either beneficial or detrimental to the risk of ruminal acidosis, depending on substrate availability, concentration, and dietary management. The synchrony of rumen-available protein and carbohydrate has been proposed as a method to increase the efficiency of microbial nitrogen production and animal productivity (Johnson, 1976) and could influence the risk of ruminal acidosis. Ammonia may “buffer” changes in rumen pH by neutralizing between 10% and 15% of the rumen VFA concentrations produced (Owens et al., 1998), a function which could reduce the risk of ruminal acidosis. Golder et al. (2014c)demonstrated that feeding canola meal to increase crude protein content produced higher ruminal ammonia concentrations and estimated metabolizable protein yield, and was associated with reduced risk of acidosis. Golder et al. (2014c) postulated that these cows would have an increase in rumen-undegradable protein availability and microbial growth. Fermenten^®^and Biochlor^®^increased *in vitro* rumen ammonia nitrogen concentrations and stimulated microbial protein nitrogen production by approximately 24.6% and 13.5%, respectively (Lean et al., 2005). Cows fed Fermenten^®^ had reduced ruminal acidosis risk *in vivo* (Golder, 2014). These findings *in toto*indicate the potential for ammonia and peptides to reduce the risk of acidosis through neutralizing VFA and increasing microbial protein production, thereby reducing the futile cycles leading to increased VFA production and energy spilling (Russell, 2007).

Overall, our findings support the concept that the rumen ecosystem is host specific and comprises a core rumen microbiota that has a unique ability to adapt to different substrates and may contribute to a host’s individual susceptibility to disorders such as ruminal acidosis (Golder et al., 2014b). Microbial interactions are extremely complex; it is a challenge to evaluate the entire microbiome and the influence of other factors, including protozoa, fungi, biofilms, and inflammatory agents, and markers requires exploration. Only a small percentage of rumen bacteria species have been characterized (McSweeney et al., 2007), highlighting the infancy of this field. Flux through metabolic pathways requires characterization, as bacteria prevalence may not reflect metabolic function. Furthermore, recognized metabolic fermentation pathways fail to explain all generated fermentation products (Hackmann et al., 2017), emphasizing a need for a continuation of microbial culturing to accompany omic technologies (Gruninger et al., 2019).

## 5 Conclusion

In summary, our findings from this multi-site, multi-country observational study, have increased the understanding of the etiology of acidosis and our hypotheses were supported. Acidosis risk was found to be associated with rumen bacterial taxa and dietary nutrient components. There were up to nine ruminal bacterial taxa associated with each acidosis risk group. The sugar and CP content of the diet are vital predictors of key ruminal bacterial taxa, and thus acidosis risk.

## Data availability statement

The datasets presented in this study can be found in Figshare (https://doi.org/10.6084/m9.figshare.22696348.v1).

## Ethics statement

The animal study was approved by the S*cibus* Animal Ethics Committee (Scibus 0618-1219), the University of California Davis Institutional Animal Care and Use Committee (protocol number #20729), the University of Wisconsin, College of Agriculture and Life Sciences Animal Care and Use Committee (approval A006225), and the Animal Care Committee at the University of Guelph (Animal Utilization Protocol 4124). The study was conducted in accordance with the local legislation and institutional requirements.

## Author contributions

HG contributed to conceptualization, sample collection, data analysis, data interpretation, and drafting of the manuscript. JR contributed to the lab work, data analysis, and data interpretation. AS contributed to data interpretation and supervision. EB contributed to data interpretation, funding, and supervision. IL contributed to conceptualization, sample collection, data analysis, data interpretation, drafting of the manuscript, and supervision. All authors contributed to the article and approved the submitted version.

## References

[B1] Abbas W. Howard J. T. Paz H. A. Hales K. E. Wells J. E. Kuehn L. A.. (2020). Influence of host genetics in shaping the rumen bacterial community in beef cattle. Sci. Rep. 10, 15101. doi: 10.1038/s41598-020-72011-9 32934296 PMC7493918

[B2] Abe K. Ueki A. Ohtaki Y. Kaku N. Watanabe K. Ueki K. (2012). *Anaerocella delicata*gen. Nov., sp. Nov., a strictly anaerobic bacterium in the phylum Bacteroidetes isolated from a methanogenic reactor of cattle farms. J. Gen. Appl. Microbiol. 58, 405–412. doi: 10.2323/jgam.58.405 23337575

[B3] Albertsen M. Hugenholtz P. Skarshewski A. Nielsen K. L. Tyson G. W. Nielsen P. H. (2013). Genome sequences of rare, uncultured bacteria obtained by differential coverage binning of multiple metagenomes. Nat. Biotechnol. 31, 533–538. doi: 10.1038/nbt.2579 23707974

[B4] Al Jassim R. A. Scott P. T. Trebbin A. L. Trott D. Pollitt C. C. (2005). The genetic diversity of lactic acid producing bacteria in the equine gastrointestinal tract. FEMS Microbiol. Lett. 248, 75–81. doi: 10.1016/j.femsle.2005.05.023 15953698

[B5] Allen M. S. (1997). Relationship between fermentation acid production in the rumen and the requirement for physically effective fiber. J. Dairy Sci. 80, 1447–1462. doi: 10.3168/jds.S0022-0302(97)76074-0 9241607

[B6] Amir A. McDonald D. Navas-Molina J. A. Kopylova E. Morton J. T. Zech Xu Z.. (2017). Deblur rapidly resolves single-nucleotide community sequence patterns. MSystems 2, e00191–e00116. doi: 10.1128/mSystems.00191-16 28289731 PMC5340863

[B7] Annison E. F. Lewis D. (1959). “Carbohydrate and volatile fatty acid metabolism,” in Metabolism in the rumen (London, UK: Methuen & Co), 59–91.

[B8] Bainbridge M. L. Cersosimo L. M. Wright A.-D. G. Kraft J. (2016). Rumen bacterial communities shift across a lactation in Holstein, Jersey and Holstein × Jersey dairy cows and correlate to rumen function, bacterial fatty acid composition and production parameters. FEMS Microbiol. Ecol. 92, 1–14. doi: 10.1093/femsec/fiw059 26985012

[B9] Bergey D. H. De Vos P. Boone D. R. Garrity G. M. Castenholz R. W. Brenner D. J.. (2011). Bergey’s manual of systematic bacteriology: Volume 3: The Firmicutes (New York: Springer).

[B10] Bokulich N. A. Subramanian S. Faith J. J. Gevers D. Gordon J. I. Knight R.. (2013). Quality-filtering vastly improves diversity estimates from Illumina amplicon sequencing. Nat. Methods 10, 57–59. doi: 10.1038/nmeth.2276 23202435 PMC3531572

[B11] Bolyen E. Rideout J. R. Dillon M. R. Bokulich N. A. Abnet C. C. Al-Ghalith G. A.. (2019). Reproducible, interactive, scalable and extensible microbiome data science using QIIME 2. Nat. Biotechnol. 37, 852–857. doi: 10.1038/s41587-019-0209-9 31341288 PMC7015180

[B12] Boone D. R. Castenholz R. W. Garrity G. M. Krieg N. R. (2011). Bergey’s manual of systematic bacteriology: Volume 4: The Bacteroidetes, Spirochaetes, Tenericutes (Mollicutes), Acidobacteria, Fibrobacteres, Fusobacteria, Dictyoglomi, Gemmatimonadetes, Lentisphaerae, Verrucomicrobia, Chlamydiae, and Planctomycetes (New York: Springer).

[B13] Bramley E. Lean I. J. Fulkerson W. J. Stevenson M. A. Rabiee A. R. Costa,. N. D. (2008). The definition of acidosis in dairy herds predominantly fed on pasture and concentrates. J. Dairy Sci. 91, 308–321. doi: 10.3168/jds.2006-601 18096953

[B14] Brede M. Orton T. Pinior B. Roch F.-F. Dzieciol M. Zwirzitz B.. (2020). Pacbio and Illumina MiSeq amplicon sequencing confirm full recovery of the bacterial community after subacute ruminal acidosis challenge in the RUSITEC system. Front. Microbiol. 11. doi: 10.3389/fmicb.2020.01813 PMC7426372 32849420

[B15] Britton R. Stock R. (1989) “Acidosis - a continual problem in cattle fed high grain diets,” in 1989 cornell nutrition conference for feed manufacturers (Ithaca, NY: Cornell University), 8–15.

[B16] Brockman R. (2005). “Glucose and short-chain fatty acid metabolism,” in Quantitative aspects of ruminant digestion and metabolism (Wallingford, UK: CABI Publishing), 291–310.

[B17] Clavel T. Lepage P. Charrier C. (2014). “The family coriobacteriaceae,” in The prokaryotes: actinobacteria . Eds. Rosenberg E. DeLong E. F. Lory S. Stackebrandt E. Thompson F. (Berlin, Heidelberg: Springer Berlin Heidelberg), 201–238.

[B18] Daghio M. Ciucci F. Buccioni A. Cappucci A. Casarosa L. Serra A.. (2021). Correlation of breed, growth performance, and rumen microbiota in two rustic cattle breeds reared under different conditions. Front. Microbiol. 12. doi: 10.3389/fmicb.2021.652031 PMC8117017 33995309

[B19] de Menezes A. B. Lewis E. O’Donovan M. O’Neill B. F. Clipson N. (2011). and doyle, E Microbiome analysis of dairy cows fed pasture or total mixed ration diets. M. FEMS Microbiol. Ecol. 78, 256–265. doi: 10.1111/j.1574-6941.2011.01151.x 21671962

[B20] Douglas A. E. (2020). The microbial exometabolome: Ecological resource and architect of microbial communities. Philos. Trans. R. Soc B: Biol. Sci. 375, 20190250. doi: 10.1098/rstb.2019.0250 PMC7133521 32200747

[B21] Enemark J. M. D. (2008). The monitoring, prevention and treatment of sub-acute ruminal acidosis (SARA): A review. Vet. J. 176, 32–43. doi: 10.1016/j.tvjl.2007.12.021 18343172

[B22] Fernando S. C. Purvis H. T. Najar F. Z. Sukharnikov L. O. Krehbiel C. R. Nagaraja T. G.. (2010). Rumen microbial population dynamics during adaptation to a high-grain diet. Appl. Environ. Microbiol. 76, 7482–7490. doi: 10.1128/AEM.00388-10 20851965 PMC2976194

[B23] Gharechahi J. Vahidi M. F. Bahram M. Han J.-L. Ding X.-Z. Salekdeh G. H. (2021). Metagenomic analysis reveals a dynamic microbiome with diversified adaptive functions to utilize high lignocellulosic forages in the cattle rumen. ISME J. 15, 1108–1120. doi: 10.1038/s41396-020-00837-2 33262428 PMC8114923

[B24] Golder H. M. (2014). Increased understandings of ruminal acidosis in dairy cattle (The University of Sydney: PhD Thesis).

[B25] Golder H. M. Celi P. Rabiee A. R. Bramley E. Lean I. J. (2012a). “Validation of an acidosis model,” in Dairy research foundation (Camden, NSW: Australia).

[B26] Golder H. M. Celi P. Rabiee A. R. Heuer C. Bramley E. Miller S. W.. (2012b). Effects of grain, fructose and histidine on ruminal pH and fermentation products during an induced subacute acidosis protocol. J. Dairy Sci. 95, 1971–1982. doi: 10.3168/jds.2011-4671 22459843

[B27] Golder H. M. Celi P. Rabiee A. R. Lean I. J. (2014a). Effects of feed additives on rumen and blood profiles during a starch and fructose challenge. J. Dairy Sci. 97, 985–1004. doi: 10.3168/jds.2013-7166 24210482

[B28] Golder H. M. Denman S. E. McSweeney C. Celi P. Lean I. J. (2014b). Ruminal bacterial community shifts in grain, sugar, and histidine challenged dairy heifers. J. Dairy Sci. 97, 5131–5150. doi: 10.3168/jds.2014-8003 24881800

[B29] Golder H. M. Denman S. E. McSweeney C. Wales W. J. Auldist M. J. Wright M. M.. (2014c). Effects of partial mixed rations and supplement amounts on milk production and composition, ruminal fermentation, bacterial communities, and ruminal acidosis. J. Dairy Sci. 97, 5763–5785. doi: 10.3168/jds.2014-8049 24997657

[B30] Golder H. LeBlanc S. Duffield T. Rossow H. Bogdanich R. Hernandez L.. (2023a). Supplementary material - characterizing ruminal acidosis risk: A multi-herd, multi-country study. J. Dairy Sci. Figshare Collection . doi: 10.6084/m9.figshare.c.6411203.v1

[B31] Golder H. M. LeBlanc S. J. Duffield T. Rossow H. A. Bogdanich R. Hernandez L.. (2023b). Characterizing ruminal acidosis risk: A multiherd, multicountry study. J. Dairy Sci. 106, 3155–3175. doi: 10.3168/jds.2022-22571 36894423

[B32] Golder H. Rossow H. Lean I. (2019). Effects of in-feed enzymes on milk production and components, reproduction, and health in dairy cows. J. Dairy Sci. 102, 8011–8026. doi: 10.3168/jds.2019-16601 31279550

[B33] Golder H. M. Thomson J. Rehberger J. Smith A. H. Block E. Lean I. J. (2023c). Associations among the genome, rumen metabolome, ruminal bacteria, and milk production in early-lactation Holsteins. J. Dairy Sci. 106, 3176–3191. doi: 10.3168/jds.2022-22573 36894426

[B34] Gruninger R. J. Ribeiro G. O. Cameron A. McAllister T. A. (2019). Invited review: Application of meta-omics to understand the dynamic nature of the rumen microbiome and how it responds to diet in ruminants. Animal 13, 1843–1854. doi: 10.1017/S1751731119000752 31062682

[B35] Guo M. Zhou Q. Zhou Y. Yang L. Liu T. Yang J.. (2014). Genomic evolution of 11 type strains within family *Planctomycetaceae* . PloS One 9, e86752. doi: 10.1371/journal.pone.0086752 24489782 PMC3906078

[B36] Hackmann T. J. Ngugi D. K. Firkins J. L. Tao J. (2017). Genomes of rumen bacteria encode atypical pathways for fermenting hexoses to short-chain fatty acids. Environ. Microbiol. 19, 4670–4683. doi: 10.1111/1462-2920.13929 28892251

[B37] Hall M. B. (2013). Dietary starch source and protein degradability in diets containing sucrose: Effects on ruminal measures and proposed mechanism for degradable protein effects. J. Dairy Sci. 96, 7093–7109. doi: 10.3168/jds.2012-5663 24054288

[B38] Harmon D. L. Britton R. A. Prior R. L. Stock R. A. (1985). Net portal absorption of lactate and volatile fatty acids in steers experiencing glucose-induced acidosis or fed a 70% concentrate diet *ad libitum* . J. Anim. Sci. 60, 560–569. doi: 10.2527/jas1985.602560x 3988634

[B39] Heldt J. S. Cochran R. C. Stokka G. L. Farmer C. G. Mathis C. P. Titgemeyer E. C.. (1999). Effects of different supplemental sugars and starch fed in combination with degradable intake protein on low-quality forage use by beef steers. J. Anim. Sci. 77, 2793–2802. doi: 10.2527/1999.77102793x 10521042

[B40] Henderson G. Cox F. Ganesh S. Jonker A. Young W. Janssen P. H. (2015). Rumen microbial community composition varies with diet and host, but a core microbiome is found across a wide geographical range. Sci. Rep. 5, 14567. doi: 10.1038/srep14567 26449758 PMC4598811

[B41] Herrera-Saldana R. Huber J. T. (1989). Influence of varying protein and starch degradabilities on performance of lactating cows. J. Dairy Sci. 72, 1477–1483. doi: 10.3168/jds.S0022-0302(89)79257-2 2760309

[B42] Hess S. Suthaus A. Melkonian M. (2016). “*Candidatus*Finniella” (*Rickettsiales, Alphaproteobacteria*), novel endosymbionts of viridiraptorid amoeboflagellates (Cercozoa, Rhizaria). Appl. Environ. Microbiol. 82, 659–670. doi: 10.1128/AEM.02680-15 26567303 PMC4711129

[B43] Hook S. E. Steele M. A. Northwood K. S. Dijkstra J. France J. Wright A.-D. G.. (2011). Impact of subacute ruminal acidosis (SARA) adaptation and recovery on the density and diversity of bacteria in the rumen of dairy cows. FEMS Microbiol. Ecol. 78, 275–284. doi: 10.1111/j.1574-6941.2011.01154.x 21692816

[B44] Hughes J. B. Hellmann J. J. Ricketts T. H. Bohannan B. J. M. (2001). Counting the uncountable: Statistical approaches to estimating microbial diversity. Appl. Environ. Microbiol. 67, 4399–4406. doi: 10.1128/aem.67.10.4399-4406.2001 11571135 PMC93182

[B45] Hungate R. E. (1966). The rumen and its microbes (New York, NY: Academic Press Inc).

[B46] International A. O. A. C. (1999). Official methods of analysis of AOAC INTERNATIONAL Washington, DC: AOAC .

[B47] Jami E. Mizrahi I. (2012). Similarity of the ruminal bacteria across individual lactating cows. Anaerobe 18, 338–343. doi: 10.1016/j.anaerobe.2012.04.003 22546373

[B48] Johnson R. R. (1976). Influence of carbohydrate solubility on non-protein nitrogen utilization in the ruminant. J. Anim. Sci. 43, 184–191. doi: 10.2527/jas1976.431184x 939724

[B49] Kamra D. N. (2005). Rumen microbial ecosystem. Curr. Sci. 89, 124–135. https://www.jstor.org/stable/24110438

[B50] Kersters K. Lisdiyanti P. Komagata K. Swings J. (2006). “The family *Acetobacteraceae*: the genera *Acetobacter, Acidomonas, Asaia, Gluconacetobacter, Gluconobacter*, and *Kozakia* ,” in The prokaryotes: A handbook on the biology of bacteria (New York: Springer), 163–200.

[B51] Khafipour E. Krause D. O. Plaizier J. C. (2009). A grain-based subacute ruminal acidosis challenge causes translocation of lipopolysaccharide and triggers inflammation. J. Dairy Sci. 92, 1060–1070. doi: 10.3168/jds.2008-1389 19233799

[B52] Kim O.-S. Cho Y.-J. Lee K. Yoon S.-H. Kim M. Na H.. (2012). Introducing EzTaxon-e: A prokaryotic 16S rRNA gene sequence database with phylotypes that represent uncultured species. Int. J. Syst. Evol. Microbiol. 62, 716–721. doi: 10.1099/ijs.0.038075-0 22140171

[B53] Klopfenstein T. J. (2001). “Distillers grains for beef cattle,” in National corn growers association, ethanol co-products workshop (Lincoln, Nebraska).

[B54] Krajcarski-Hunt H. Plaizier J. C. Walton J. P. Spratt R. McBride B. W. (2002). Short communication: Effect of subacute ruminal acidosis on in *situ*fiber digestion in lactating dairy cows. J. Dairy Sci. 85, 570–573. doi: 10.3168/jds.S0022-0302(02)74110-6 11949861

[B55] Krieg N. R. (2010). “Bacteroidales ord. Nov,” in Bergey’s manual of systematics of archaea and bacteria , vol. 25. (New York: Springer).

[B56] Lean I. J. Golder H. M. Hall M. B. (2014). Feeding, evaluating, and controlling rumen function. Vet. Clin. N. Am.-Food A 30, 539–575. doi: 10.1016/j.cvfa.2014.07.003 25249402

[B57] Lean I. J. Webster T. K. M. Hoover W. Chalupa W. Sniffen C. J. Evans E.. (2005). Effects of BioChlor and Fermenten on microbial protein synthesis in continuous culture fermenters. J. Dairy Sci. 88, 2524–2536. doi: 10.3168/jds.S0022-0302(05)72930-1 15956315

[B58] Leng R. (2018). Unravelling methanogenesis in ruminants, horses and kangaroos: The links between gut anatomy, microbial biofilms and host immunity. Anim. Prod. Sci. 58, 1175–1191. doi: 10.1071/AN15710

[B59] Lepš J. Šmilauer P. (2003). Multivariate analysis of ecological data using CANOCO (Cambridge, United Kingdom: Cambridge University Press). doi: 10.1017/CBO9780511615146

[B60] Mao S. Zhang R. Wang D. Zhu W. (2013). Impact of subacute ruminal acidosis (SARA) adaptation on rumen microbiota in dairy cattle using pyrosequencing. Anaerobe 24, e19. doi: 10.1016/j.anaerobe.2013.08.003 23994204

[B61] Marchandin H. Teyssier C. Campos J. Jean-Pierre H. Roger F. Gay B.. (2010). *Negativicoccus succinicivorans*gen. Nov., sp. Nov., isolated from human clinical samples, emended description of the family *Veillonellaceae*and description of *Negativicutes classis*nov., *Selenomonadales*ord. Nov. And *Acidaminococcaceae*fam. Nov. In the bacterial phylum *Firmicutes* . Int. J. Syst. Evol. Microbiol. 60, 1271–1279. doi: 10.1099/ijs.0.013102-0 19667386

[B62] McCann J. C. Luan S. Cardoso F. C. Derakhshani H. Khafipour E. (2016). Induction of subacute ruminal acidosis affects the ruminal microbiome and epithelium. Loor J. J. Front. Microbiol. 7. doi: 10.3389/fmicb.2016.00701 PMC4870271 27242724

[B63] McSweeney C. Denman S. Wright A. Yu Z. (2007). Application of recent DNA/RNA-based techniques in rumen ecology. Asian Australas. J. Anim. Sci. 20, 283–294. doi: 10.5713/ajas.2007.283

[B64] Mertens D. R. (1997). Creating a system for meeting the fiber requirements of dairy cows. J. Dairy Sci. 80, 1463–1481. doi: 10.3168/jds.S0022-0302(97)76075-2 9241608

[B65] Morotomi M. Nagai F. Watanabe Y. (2012). Description of *christensenella minuta*gen. Nov., sp. Nov., isolated from human faeces, which forms a distinct branch in the order *clostridiales*, and proposal of *christensenellaceae*fam. Nov. Int. J. Syst. Evol. Microbiol. 62, 144–149. doi: 10.1099/ijs.0.026989-0 21357455

[B66] Morris J. J. Lenski R. E. Zinser E. R. (2012). The black queen hypothesis: Evolution of dependencies through adaptive gene loss. MBio 3, e00036–e00012. doi: 10.1128/mBio.00036-12 22448042 PMC3315703

[B67] Nagaraja T. G. Titgemeyer E. C. (2007). Ruminal acidosis in beef cattle: The current microbiological and nutritional outlook. J. Dairy Sci. 90, E17–E38. doi: 10.3168/jds.2006-478 17517750

[B68] Nasrollahi S. Zali A. Ghorbani G. Shahrbabak M. M. Abadi M. H. S. (2017). Variability in susceptibility to acidosis among high producing mid-lactation dairy cows is associated with rumen pH, fermentation, feed intake, sorting activity, and milk fat percentage. Anim. Feed Sci. Technol. 228, 72–82. doi: 10.1016/j.anifeedsci.2017.03.007

[B69] Nocek J. E. (1997). Bovine acidosis: Implications on laminitis. J. Dairy Sci. 80, 1005–1028. doi: 10.3168/jds.S0022-0302(97)76026-0 9178142

[B70] Oetzel G. R. (2003). Subacute ruminal acidosis in dairy cattle. Adv. Dairy Technol. 15, 307–317.

[B71] O’Grady L. Doherty M. L. Mulligan F. J. (2008). Subacute ruminal acidosis (SARA) in grazing Irish dairy cows. Vet. J. 176, 44–49. doi: 10.1016/j.tvj1.2007.12.017 18328751

[B72] Ormerod K. L. Wood D. L. Lachner N. Gellatly S. L. Daly J. N. Parsons J. D.. (2016). Genomic characterization of the uncultured *Bacteroidales*family *S24-7*inhabiting the guts of homeothermic animals. Microbiome 4, 1–17. doi: 10.1186/s40168-016-0181-2 27388460 PMC4936053

[B73] Ouyang J. Wang M. Bu D. Ma L. Liu F. Xue C.. (2021). Ruminal microbes exhibit a robust circadian rhythm and are sensitive to melatonin. Front. Nutr. 8. doi: 10.3389/fnut.2021.760578 PMC8573100 34760910

[B74] Owens F. N. Secrist D. S. Hill W. J. Gill D. R. (1998). Acidosis in cattle: A review. J. Anim. Sci. 76, 275–286. doi: 10.2527/1998.761275x 9464909

[B75] Petri R. M. Schwaiger T. Penner G. B. Beauchemin K. A. Forster R. J. McKinnon J. J.. (2013). Characterization of the core rumen microbiome in cattle during transition from forage to concentrate as well as during and after an acidotic challenge. PloS One 8, e83424. doi: 10.1371/journal.pone.0083424 24391765 PMC3877040

[B76] Plaizier J. C. Krause D. O. Gozho G. N. McBride B. W. (2008). Subacute ruminal acidosis in dairy cows: The physiological causes, incidence and consequences. Vet. J. 176, 21–31. doi: 10.1016/j.tvj1.2007.12.016 18329918

[B77] Plaizier J. C. Li S. Danscher A. M. Derakshani H. Andersen P. H. Khafipour E. (2017). Changes in microbiota in rumen digesta and feces due to a grain-based subacute ruminal acidosis (SARA) challenge. Microb. Ecol. 1-11. doi: 10.1007/s00248-017-0940-z 28175972

[B78] Plaizier J. C. Mesgaran D. Derakhshani H. Golder H. Khafipour E. Kleen J.. (2018). Invited review: Enhancing gut health in dairy cows. Animal 12(Suppl 2), s399–s418. doi: 10.1017/S1751731118001921 30139397

[B79] Plaizier J. Mulligan F. Neville E. Guan L. Steele M. Penner G. (2022). Invited review: Effect of subacute ruminal acidosis on gut health of dairy cows. J. Dairy Sci. 105, 7141–7160. doi: 10.3168/jds.2022-21960 35879171

[B80] Pollock J. Glendinning L. Wisedchanwet T. Watson M. (2018). The madness of microbiome: Attempting to find consensus “best practice” for 16s microbiome studies. Appl. Environ. Microbiol. 84, e02627–e02617. doi: 10.1128/AEM.02627-17 29427429 PMC5861821

[B81] Rognes T. Flouri T. Nichols B. Quince C. Mahé F. (2016). VSEARCH: A versatile open source tool for metagenomics. PeerJ 4, e2584. doi: 10.7717/peerj.2584 27781170 PMC5075697

[B82] Russell J. B. (2007). The energy spilling reactions of bacteria and other organisms. J. Mol. Microbiol. Biotechnol. 13, 1–11. doi: 10.1159/000103591 17693707

[B83] Saleem F. Ametaj B. N. Bouatra S. Mandal R. Zebeli Q. Dunn S. M.. (2012). A metabolomics approach to uncover the effects of grain diets on rumen health in dairy cows. J. Dairy Sci. 95, 6606–6623. doi: 10.3168/jds.2012-5403 22959937

[B84] Shabat S. K. B. Sasson G. Doron-Faigenboim A. Durman T. Yaacoby S. Berg Miller M. E.. (2016). Specific microbiome-dependent mechanisms underlie the energy harvest efficiency of ruminants. ISME J. 10, 2958–2972. doi: 10.1038/ismej.2016.62 27152936 PMC5148187

[B85] Siewert C. Hess W. R. Duduk B. Huettel B. Reinhardt R. Büttner C.. (2014). Complete genome determination and analysis of *Acholeplasma oculi*strain 19L, highlighting the loss of basic genetic features in the *Acholeplasmataceae* . BMC Genomics 15, 1–16. doi: 10.1186/1471-2164-15-931 25344468 PMC4221730

[B86] Sniffen C. J. O’Connor J. D. Van Soest P. J. Fox D. G. Russell J. B. (1992). A net carbohydrate and protein system for evaluating cattle diets: II. Carbohydrate and protein availability. J. Anim. Sci. 70, 3562–3577. doi: 10.2527/1992.70113562x 1459919

[B87] Solden L. M. Hoyt D. W. Collins W. B. Plank J. E. Daly R. A. Hildebrand E.. (2017). New roles in hemicellulosic sugar fermentation for the uncultivated Bacteroidetes family BS11. ISME J. 11, 691–703. doi: 10.1038/ismej.2016.150 27959345 PMC5322302

[B88] Spring S. Bunk B. Spröer C. Schumann P. Rohde M. Tindall B. J.. (2016). Characterization of the first cultured representative of Verrucomicrobia subdivision 5 indicates the proposal of a novel phylum. ISME J. 10, 2801–2816. doi: 10.1038/ismej.2016.84 27300277 PMC5148204

[B89] Stewart C. S. Flint J. F. Bryant M. P. (1997). “The rumen bacteria,” in The rumen microbial ecosystem , 2nd ed. Eds. Hobson P. N. Stewart C. S.(London, UK: Blackie Academic and Professional), 10–72.

[B90] Supelco (1975). GC separation of VFA C2-C5. Bulletin 749 (Bellefonte, PA: Supelco Inc.).

[B91] Tajima K. Arai S. Ogata K. Nagamine T. Matsui H. Nakamura M.. (2000). Rumen bacterial community transition during adaptation to high-grain diet. Anaerobe 6, 273–284. doi: 10.1006/anae.2000.0353

[B92] Taxis T. M. Wolff S. Gregg S. J. Minton N. O. Zhang C. Dai J.. (2015). The players may change but the game remains: Network analyses of ruminal microbiomes suggest taxonomic differences mask functional similarity. Nucleic Acids Res. 43, 9600–9612. doi: 10.1093/nar/gkv973 26420832 PMC4787786

[B93] Todar K. (2012). Nutrition and growth of bacteria in todar’s online textbook on bacteriology. Available at: http://textbookofbacteriology.net/nutgro.html (Accessed 27 Nov 2013).

[B94] Walters W. Hyde E. R. Berg-Lyons D. Ackermann G. Humphrey G. Parada A.. (2016). Improved bacterial 16S rRNA gene (V4 and V4-5) and fungal internal transcribed spacer marker gene primers for microbial community surveys. mSystems 1, e00009–e00015. doi: 10.1128/mSystems.00009-15 PMC5069754 27822518

[B95] Wiegand S. Jogler M. Jogler C. (2018). On the maverick planctomycetes. FEMS Microbiol. Rev. 42, 739–760. doi: 10.1093/femsre/fuy029 30052954

[B96] Williams K. P. Gillespie J. J. Sobral B. W. Nordberg E. K. Snyder E. E. Shallom J. M.. (2010). Phylogeny of gammaproteobacteria. J. Bacteriol. 192, 2305–2314. doi: 10.1128/JB.01480-09 20207755 PMC2863478

[B97] Xue M. Sun H. Wu X. Liu J. (2018). Assessment of rumen microbiota from a large dairy cattle cohort reveals the pan and core bacteriomes contributing to varied phenotypes. Appl. Environ. Microbiol. 84, e00970–e00918. doi: 10.1128/AEM.00970-18 30054362 PMC6146982

[B98] Yamada T. Sekiguchi Y. Hanada S. Imachi H. Ohashi A. Harada H.. (2006). *Anaerolinea thermolimosa*sp. Nov., *levilinea saccharolytica*gen. Nov., sp. Nov. And *leptolinea tardivitalis*gen. Nov., sp. Nov., novel filamentous anaerobes, and description of the new classes *anaerolineae classis*nov. And *caldilineae*classis nov. In the bacterial phylum *chloroflexi* . Int. J. Syst. Evol. Microbiol. 56, 1331–1340. doi: 10.1099/ijs.0.64169-0 16738111

[B99] Zebeli Q. Aschenbach J. R. Tafaj M. Boguhn J. Ametaj B. N. Drochner W. (2012). Invited review: Role of physically effective fiber and estimation of dietary fiber adequacy in high-producing dairy cattle. J. Dairy Sci. 95, 1041–1056. doi: 10.3168/jds.2011-4421 22365188

