# Supplementary material for: Ruminal bacterial communities differ in early-lactation dairy cows with differing risk of ruminal acidosis
Source: Front Microbiomes. 2023 Sep 29;2:1212255. doi: 10.3389/frmbi.2023.1212255 (PMC12993616; doi:10.3389/frmbi.2023.1212255)
Supplement: Supplementary file 1 [file Table_1.docx]

Supplementary Material

Ruminal bacterial communities differ in early lactation dairy cows with differing risk of ruminal acidosis

Helen Marie Golder^*^, Josh Rehberger, Alexandra Helena Smith, Elliot Block, Ian John Lean

*** Correspondence:** Helen Golder: heleng@scibus.com.au

**Supplementary Table 1.** The x and y co-ordinates and Cfit2 values for the 20 bacterial families with best fit to acidosis risk group only, region only, or acidosis risk group, region, and dietary nutrients in redundancy analysis biplots produced by Canoco5 (Microcomputer Power, Ithaca, NY). The total number of bacterial families included in the table is 29 as 11 were in the 20 best fit list for both acidosis group and region.

| Family | Acidosis group (G) | | | Region (R) | | | G, R & dietary nutrients | | |
| --- | --- | --- | --- | --- | --- | --- | --- | --- | --- |
|  | X | Y | Cfit2 | X | Y | Cfit2 | X | Y | Cfit2 |
| Gammaproteobacteria_c | -0.496 | -0.073 | 0.251 | -0.281 | -0.369 | 0.215 | -0.480 | -0.337 | 0.344 |
| Lachnospiraceae | 0.497 | -0.065 | 0.251 | 0.435 | 0.105 | 0.200 | 0.606 | 0.126 | 0.383 |
| Erysipelotrichaceae | 0.496 | 0.064 | 0.250 | 0.163 | 0.564 | 0.345 | 0.402 | 0.512 | 0.424 |
| EF436358_f | -0.466 | -0.033 | 0.218 |  |  |  |  |  |  |
| Christensenellaceae | -0.441 | 0.122 | 0.209 | 0.249 | -0.458 | 0.272 | -0.060 | -0.675 | 0.459 |
| AF050559_f | -0.381 | 0.218 | 0.193 |  |  |  |  |  |  |
| Planctomycetaceae | -0.421 | 0.016 | 0.178 |  |  |  | -0.114 | -0.471 | 0.235 |
| AY244965_f | -0.415 | 0.012 | 0.172 | -0.351 | -0.206 | 0.166 | -0.458 | -0.178 | 0.242 |
| Veillonellaceae | 0.370 | 0.014 | 0.137 |  |  |  |  |  |  |
| GU304534_f | -0.349 | -0.105 | 0.133 | -0.388 | -0.239 | 0.208 |  |  |  |
| Acholeplasmataceae | -0.347 | 0.072 | 0.125 | -0.430 | -0.078 | 0.191 | -0.503 | -0.031 | 0.254 |
| EF445272_f | -0.325 | 0.134 | 0.123 |  |  |  | -0.117 | -0.483 | 0.247 |
| Coriobacteriaceae | 0.350 | 0.009 | 0.122 | 0.508 | 0.172 | 0.288 | 0.582 | 0.051 | 0.341 |
| RF16_f | -0.293 | 0.174 | 0.116 |  |  |  | -0.400 | -0.239 | 0.217 |
| Anaerocella_f | 0.317 | -0.036 | 0.102 |  |  |  | 0.048 | 0.456 | 0.210 |
| Paracaedibacteraceae | -0.288 | -0.075 | 0.089 |  |  |  |  |  |  |
| Ruminococcaceae | -0.287 | 0.016 | 0.083 |  |  |  |  |  |  |
| BS11_f | -0.261 | -0.121 | 0.083 | -0.336 | -0.284 | 0.193 |  |  |  |
| Acetobacteraceae | -0.215 | -0.183 | 0.080 | -0.253 | -0.472 | 0.287 |  |  |  |
| Eubacteriaceae | 0.258 | 0.088 | 0.074 | -0.018 | 0.485 | 0.235 | 0.143 | 0.439 | 0.213 |
| Prevotellaceae |  |  |  | -0.574 | 0.243 | 0.388 | -0.495 | 0.403 | 0.407 |
| Streptococcaceae |  |  |  | 0.531 | -0.010 | 0.282 | 0.521 | -0.215 | 0.318 |
| Anaerolinaceae |  |  |  | 0.268 | -0.423 | 0.251 | 0.097 | -0.533 | 0.293 |
| Acidaminococcaceae |  |  |  | -0.487 | 0.035 | 0.238 | -0.450 | 0.222 | 0.252 |
| Carnobacteriaceae |  |  |  | 0.466 | -0.141 | 0.237 | 0.450 | -0.262 | 0.271 |
| S247_f |  |  |  | -0.479 | 0.072 | 0.235 | -0.375 | 0.265 | 0.211 |
| Leuconostocaceae |  |  |  | 0.335 | -0.338 | 0.227 | 0.245 | -0.393 | 0.214 |
| Lactobacillaceae |  |  |  | 0.378 | -0.257 | 0.209 |  |  |  |
| Saccharimonadaceae |  |  |  | 0.270 | -0.314 | 0.171 | 0.089 | -0.478 | 0.237 |
