# Supplementary material for: Ruminal bacterial communities differ in early-lactation dairy cows with differing risk of ruminal acidosis
Source: Front Microbiomes. 2023 Sep 29;2:1212255. doi: 10.3389/frmbi.2023.1212255 (PMC12993616; doi:10.3389/frmbi.2023.1212255)
Supplement: Supplementary file 2 [file Table_2.docx]

Supplementary Material

Ruminal bacterial communities differ in early lactation dairy cows with differing risk of ruminal acidosis

Helen Marie Golder^*^, Josh Rehberger, Alexandra Helena Smith, Elliot Block, Ian John Lean

*** Correspondence:** Helen Golder: heleng@scibus.com.au

**Supplementary Table 2.** The coefficient, SE, and significance for diet nutrient components from a backwards stepwise elimination regression, that included the fixed effect of region, for the 26 phyla that had at least one sample with a relative abundance of >0.3% which include the 9 phyla of interest included in Table 6. Nutrients with a significance *P* <0.100 remained in the model, except for confounding. The root mean square error (RMSE) for each model (phyla) is given, abundance [relative and center logged ratio (CLR)] for each phylum, and whether the phylum was a phylum of interest already included in Table 6.

| Phylum | Coefficient | SE | *P*-value | RMSE | Mean abundance | | In Table 6 |
| --- | --- | --- | --- | --- | --- | --- | --- |
|  |  |  |  |  | Relative (%) | CLR |  |
| Acidobacteria |  |  |  | 0.340 | <0.001 | -0.005 | No |
| CP | -0.037 | 0.023 | 0.104 |  |  |  |  |
| Actinobacteria |  |  |  | 0.942 | 8.69 | -0.014 | Yes |
| CP | 0.114 | 0.064 | 0.077 |  |  |  |  |
| Sugar | 0.146 | 0.080 | 0.068 |  |  |  |  |
| NDF | 0.049 | 0.028 | 0.084 |  |  |  |  |
| Starch | 0.089 | 0.043 | 0.040 |  |  |  |  |
| Crude fat | 0.237 | 0.120 | 0.048 |  |  |  |  |
| Armatimonadetes |  |  |  | 0.496 | 0.01 | -0.004 | No |
| Crude fat | -0.072 | 0.049 | 0.141 |  |  |  |  |
| Bacteroidetes |  |  |  | 0.697 | 28.6 | 0.000 | Yes |
| Sugar | -0.085 | 0.048 | 0.080 |  |  |  |  |
| NDF | -0.009 | 0.013 | 0.480 |  |  |  |  |
| Chlamydiae |  |  |  | 0.104 | <0.001 | 0.002 | No |
| NDF | -0.003 | 0.003 | 0.308 |  |  |  |  |
| Chloroflexi |  |  |  | 1.564 | 0.48 | 0.000 | No |
| Crude fat | -0.117 | 0.114 | 0.303 |  |  |  |  |
| Cu922841P |  |  |  | 0.061 | <0.001 | 0.000 |  |
| CP | 0.019 | 0.004 | <0.001 |  |  |  |  |
| Cyanobacteria |  |  |  | 1.184 | 0.15 | -0.004 | No |
| CP | -0.221 | 0.073 | 0.002 |  |  |  |  |
| Sugar | -0.313 | 0.092 | 0.001 |  |  |  |  |
| Deinococcusthermus |  |  |  | 0.053 | <0.001 | -0.001 | No |
| CP | 0.014 | 0.003 | 0.000 |  |  |  |  |
| Eu844128P |  |  |  | 0.279 | <0.001 | 0.004 | No |
| Starch | -0.017 | 0.007 | 0.018 |  |  |  |  |
| Elusimicrobia |  |  |  | 0.417 | 0.01 | -0.006 | No |
| NDF | 0.015 | 0.010 | 0.150 |  |  |  |  |
| Fibrobacteres |  |  |  | 1.185 | 0.06 | -0.023 | Yes |
| Sugar | -0.157 | 0.088 | 0.075 |  |  |  |  |
| Firmicutes |  |  |  | 0.198 | 50.5 | 0.000 | Yes |
| Sugar | 0.028 | 0.015 | 0.068 |  |  |  |  |
| Crude fat | 0.023 | 0.016 | 0.163 |  |  |  |  |
| Fusobacteria |  |  |  | 0.286 | 0.01 | 0.009 | No |
| NDF | 0.021 | 0.008 | 0.006 |  |  |  |  |
| Starch | 0.023 | 0.012 | 0.043 |  |  |  |  |
| Crude fat | 0.051 | 0.034 | 0.134 |  |  |  |  |
| Gemmatimonadetes |  |  |  | 0.146 | <0.001 | 0.003 | No |
| CP | 0.013 | 0.010 | 0.197 |  |  |  |  |
| NDF | 0.012 | 0.004 | 0.004 |  |  |  |  |
| Starch | 0.009 | 0.005 | 0.080 |  |  |  |  |
| Lentisphaerae |  |  |  | 1.505 | 0.61 | -0.007 | Yes |
| CP | -0.166 | 0.096 | 0.083 |  |  |  |  |
| Sugar | -0.288 | 0.108 | 0.008 |  |  |  |  |
| Peregrinibacteria |  |  |  |  | <0.001 | 0.013 | No |
| Starch | 0.021 | 0.007 | 0.005 |  |  |  |  |
| Crude fat | 0.048 | 0.024 | 0.049 |  |  |  |  |
| Planctomycetes |  |  |  | 1.150 | 0.67 | -0.019 | Yes |
| CP | -0.214 | 0.074 | 0.004 |  |  |  |  |
| Proteobacteria |  |  |  | 0.569 | 3.61 | 0.017 | Yes |
| CP | -0.068 | 0.036 | 0.058 |  |  |  |  |
| Sugar | -0.168 | 0.042 | <0.001 |  |  |  |  |
| NDF | -0.034 | 0.014 | 0.013 |  |  |  |  |
| Saccharibacteria |  |  |  | 0.962 | 0.16 | 0.051 | No |
| CP | -0.251 | 0.059 | <0.001 |  |  |  |  |
| Crude fat | -0.150 | 0.081 | 0.065 |  |  |  |  |
| Spirochaetes |  |  |  | 1.603 | 1.41 | 0.018 | Yes |
| Sugar | -0.364 | 0.130 | 0.005 |  |  |  |  |
| Starch | -0.098 | 0.061 | 0.112 |  |  |  |  |
| Crude fat | -0.445 | 0.208 | 0.032 |  |  |  |  |
| SR1 |  |  |  | 1.481 | 0.28 | 0.069 | No |
| CP | -0.164 | 0.098 | 0.093 |  |  |  |  |
| Sugar | -0.290 | 0.119 | 0.015 |  |  |  |  |
| Synergistetes |  |  |  | 0.847 | 0.02 | -0.006 | No |
| CP | -0.097 | 0.054 | 0.074 |  |  |  |  |
| Tenericutes |  |  |  | 0.332 | 4.62 | 0.016 | Yes |
| CP | -0.099 | 0.021 | 0.000 |  |  |  |  |
| Crude fat | -0.067 | 0.033 | 0.040 |  |  |  |  |
| TDNP |  |  |  | 0.263 | <0.001 | -0.006 | No |
| Sugar | -0.048 | 0.020 | 0.017 |  |  |  |  |
| Starch | -0.026 | 0.010 | 0.009 |  |  |  |  |
| Crude fat | -0.084 | 0.033 | 0.012 |  |  |  |  |
| Verrucomicrobia |  |  |  | 0.846 | 0.03 | 0.013 | No |
| Starch | -0.017 | 0.025 | 0.511 |  |  |  |  |

CP = crude protein; NDF = neutral detergent fiber
