# Supplementary material for: Ruminal bacterial communities differ in early-lactation dairy cows with differing risk of ruminal acidosis
Source: Front Microbiomes. 2023 Sep 29;2:1212255. doi: 10.3389/frmbi.2023.1212255 (PMC12993616; doi:10.3389/frmbi.2023.1212255)
Supplement: Supplementary file 3 [file Image_1.pdf]

Supplementary Material

Ruminal bacterial communities differ in early lactation dairy cows with differing risk of ruminal acidosis

Helen Marie Golder*, Josh Rehberger, Alexandra Helena Smith, Elliot Block, Ian John Lean

*** Correspondence:** Helen Golder: heleng@scibus.com.au

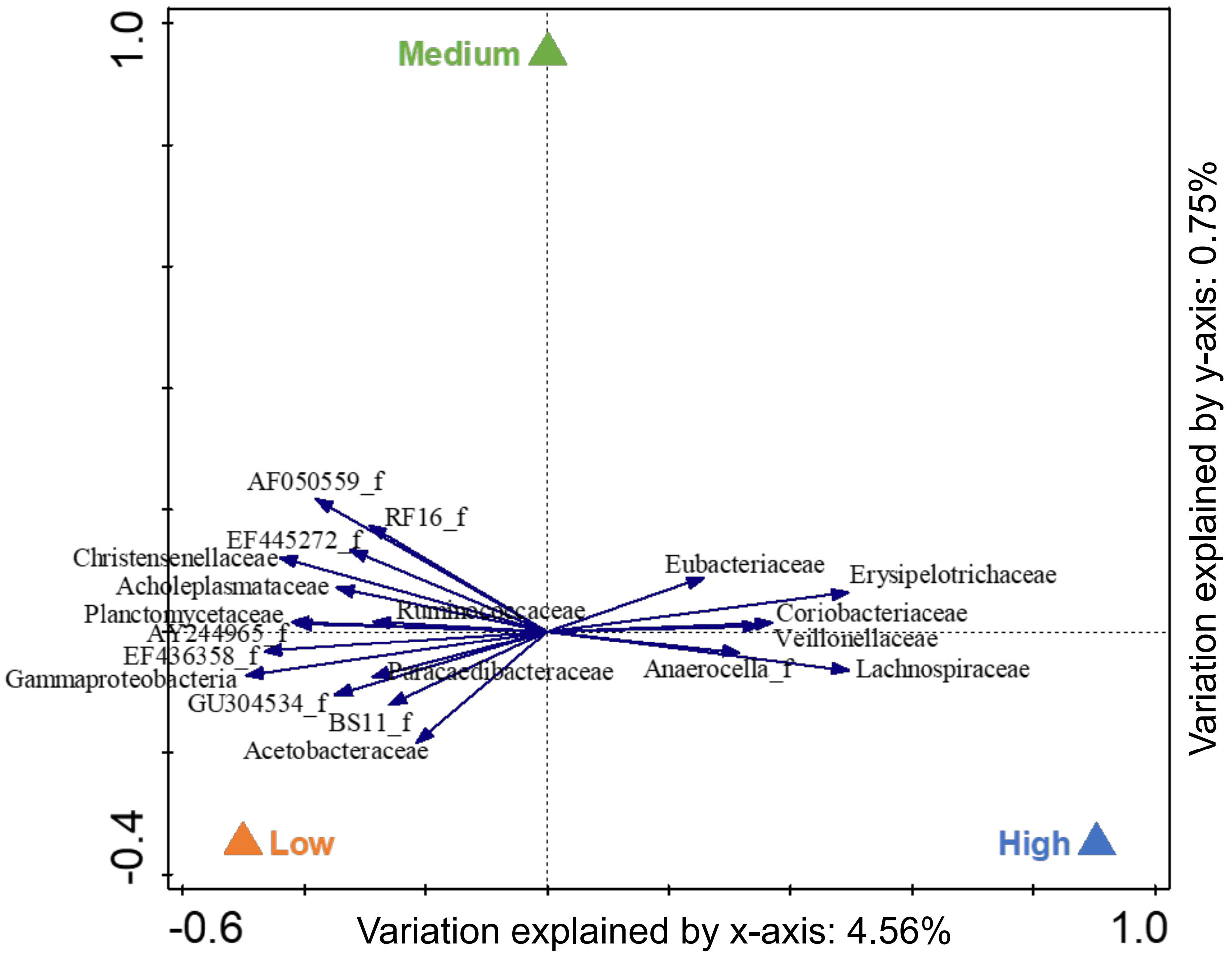

Supplementary Figure 1. Correlation biplot of the redundancy analysis of the center log transformed relative abundance of bacterial families (blue arrows) with respect to acidosis risk group (triangles). The triangle is the midpoint of the samples in that group. The 20 bacterial families with the best fit to acidosis risk group are displayed where the length of the arrows are approximate correlation coefficients between the bacterial families and acidosis risk groups with relative abundance increasing in the direction of the arrow. The total variation associated with acidosis risk group is 5.3%.
